# *Candida albicans* Sfl1/Sfl2 regulatory network drives the formation of pathogenic microcolonies

**DOI:** 10.1371/journal.ppat.1007316

**Published:** 2018-09-25

**Authors:** Andrew D. McCall, Rohitashw Kumar, Mira Edgerton

**Affiliations:** Department of Oral Biology, School of Dental Medicine, The State University of New York at Buffalo, Buffalo, New York, United States of America; University of Toronto, CANADA

## Abstract

*Candida albicans* is an opportunistic fungal pathogen that can infect oral mucosal surfaces while being under continuous flow from saliva. Under specific conditions, *C*. *albicans* will form microcolonies that more closely resemble the biofilms formed *in vivo* than standard *in vitro* biofilm models. However, very little is known about these microcolonies, particularly genomic differences between these specialized biofilm structures and the traditional *in vitro* biofilms. In this study, we used a novel flow system, in which *C*. *albicans s*pontaneously forms microcolonies, to further characterize the architecture of fungal microcolonies and their genomics compared to non-microcolony conditions. Fungal microcolonies arose from radially branching filamentous hyphae that increasingly intertwined with one another to form extremely dense biofilms, and closely resembled the architecture of *in vivo* oropharyngeal candidiasis. We identified 20 core microcolony genes that were differentially regulated in flow-induced microcolonies using RNA-seq. These genes included *HWP1, ECE1, IHD1*, *PLB1*, *HYR1*, *PGA10*, and *SAP5*. A predictive algorithm was utilized to identify ten transcriptional regulators potentially involved in microcolony formation. Of these transcription factors, we found that Rob1, Ndt80, Sfl1 and Sfl2, played a key role in microcolony formation under both flow and static conditions and to epithelial surfaces. Expression of core microcolony genes were highly up-regulated in Δ*sfl1* cells and down-regulated in both Δ*sfl2* and Δ*rob1* strains. Microcolonies formed on oral epithelium using *C*. *albicans* Δ*sfl1*, Δ*sfl2* and Δ*rob1* deletion strains all had altered adhesion, invasion and cytotoxicity. Furthermore, epithelial cells infected with deletion mutants had reduced (*SFL2*, *NDT80*, and *ROB1*) or enhanced (*SFL2*) immune responses, evidenced by phosphorylation of MKP1 and c-Fos activation, key signal transducers in the hyphal invasion response. This profile of microcolony transcriptional regulators more closely reflects Sfl1 and Sfl2 hyphal regulatory networks than static biofilm regulatory networks, suggesting that microcolonies are a specialized pathogenic form of biofilm.

## Author summary

*Candida albicans* is an opportunistic fungal pathogen that can infect many parts of the body, including the oral cavity, where saliva continuously flows across exposed surfaces. However, only a handful of studies have looked at growth under flow, and many questions about how *C*. *albicans* grows in these conditions remain unanswered. In our previous study, we found that *C*. *albicans* grown on plastic slides and with continuous flow will spontaneously form distinct specialized biofilm structures termed microcolonies. The structure of these microcolonies closely resembles *C*. *albicans* plaques found within the oral cavity during mucosal infection. Here, we investigated the genes and genetic regulators that play a role in microcolony formation, and which of these genetic factors activate an early response in the host. We determined that 20 *C*. *albicans* genes make up the core microcolony genes, with many playing key roles in the formation of these structures. We also discovered six gene regulators of microcolony formation. Each of these regulators also plays a role in the ability of these microcolonies to adhere and invade into oral epithelial cells.

## Introduction

*Candida albicans* is a polymorphic fungal pathogen and is an opportunistic causative agent of oropharyngeal candidiasis (OPC) [1]. In oral mucosal disease, *C*. *albicans* cells adhere to epithelium and form surface plaques characterized by dense interlocking hyphae. *In vivo* plaques recovered from tongues of infected mice consist of discrete patches of cells with multiple hyphae extending from the central mass in a starburst shape [2]. We found that the length and density of hyphae was dependent upon Sap6 expression, and that this phenotype was well reproduced by *C*. *albicans* microcolonies formed *in vitro* on solid surfaces using hyphal-inducing medium (RPMI, serum or Spider) and CO_2_. Furthermore, *in vivo* plaques with more extensive filamentation and hyphal length also had higher levels of Mkc1 and Cek1 MAPKinase phosphorylation [2], indicating that morphogenic signaling processes are integral to microcolony development. Thus, *C*. *albicans* microcolonies represent a specific, and potentially specialized, form of biofilm growth whose function is not known.

Many bacteria form microcolonies on surfaces *in vitro*; however their architecture is quite different from fungal microcolonies in that bacteria form mushroom-shaped single cell aggregates containing water channels needed for success of the biofilm [3]. Although “microcolony” has been used to describe an aggregated cluster of yeast cells in the early stages of biofilm formation [4]; a better descriptor for fungal microcolonies that we use here is radiating hyphal biofilms formed on surfaces. Several other filamentous yeasts, including *Aspergillus fumigatus* [5,6], have previously been reported to form microcolonies. During microcolony formation, *C*. *albicans* generates a unique architecture consisting of hyphae growing radially outwards from a central mother cell. These hyphae further branch as they continue to grow, and ultimately form a dense web of intertwined hyphae that reach confluence in a mature biofilm [6]. Until now, *C*. *albicans* microcolonies formation was induced using medium specific to hyphal formation at 37°C and with CO_2_. However, we recently discovered that *C*. *albicans* form robust microcolonies in a flow system at 37°C without CO_2_ and in non-hyphal inducing medium [7].

*C*. *albicans* microcolonies have a fundamentally different structure than biofilms formed under static conditions. Static biofilms grown with Spider medium at 37°C are initiated by adherent yeast cells that form a basal layer, and only later form germ tubes projecting upwards away from the surface [4]. *In vitro* static biofilms have been a primary model used for studying genes and transcriptional regulators involved in biofilm formation, although they differ significantly from non-static models, as illustrated by differences in architecture of biofilms formed with a rat catheter model (a flow model) [8,9]. Nevertheless, many studies have identified critical genes essential for biofilm formation using static systems [10–12]. Many of these essential genes are involved in hyphae formation, as illustrated by the reduction in adhesion and biofilm formation in *C*. *albicans* mutants defective in hyphae formation [13]. Studies of transcriptional regulators (TRs) have further illustrated the importance of hyphae formation and adhesion in biofilm formation. To date, nine master regulators of biofilm formation have been discovered including Brg1, Rob1, Ndt80, Tec1, Efg1, Bcr1, Gal4, Flo8, and Rfx2 [11,12]. Nearly all these regulators (except Gal4) have a demonstrated role in hyphae formation [14–21], and several of them (Efg1, Tec1, Bcr1, and Rfx2) also play a role in adhesion [18,21–23]. Numerous other transcriptional regulators have also been shown to modulate morphological transition of *C*. *albicans* including Mcm1, Nrg1, Tye7, Rca1, Fkh2, Sfl1, and Sfl2 [24–29]. Also, Sfl1 and Sfl2 both associate *in vivo* with Efg1 and Ndt80, and antagonistically control *C*. *albicans* morphogenesis in response to temperature by modulation of the expression of key regulators of hyphal growth [29]. These data suggest that Sfl1 and Sfl2 act as the central on-off switch for hyphal formation and expression of hyphal specific genes associated with virulence. Since microcolonies have extensive hyphal architecture, our premise was that *SFL1* and *SFL2* are also likely to be central regulators of microcolony morphogenesis, and we expected to find a transcriptomic profile of microcolonies consistent with genes regulated by this complex.

A striking finding of *in vitro* expression profiling of *C*. *albicans* Sfl1/Sfl2 hyphal growth is that genes involved in biofilm formation, adhesion and virulence are over-represented [10,29]. However, little is known about whether *SFL1* and *SFL2* morphogenesis regulators also impact *in vivo* formation of *C*. *albicans* biofilms on oral mucosal epithelium. *C*. *albicans* hyphae are required for production of pro-inflammatory cytokines upon infection of human epithelial cell lines. Hyphal invasion resulted in phosphorylation of the MAPK, MKP1 in an ERK1/2 dependent manner, as well as the c-Fos activation [30]. In contrast, yeast cells of *C*. *albicans* activated only the NF-κB subunit p65 and the early c-Jun pathway, but ultimately did not result in cytokine production [30]; showing that epithelial cells primarily respond to *C*. *albicans* hyphae. Thus, we expect that infection of epithelial cells with *C*. *albicans* microcolonies will elicit a robust pro-inflammatory response due to their extensive hyphae, and that Sfl1 and Sfl2 will be central regulators of this response.

To gain a better understanding of flow-induced microcolonies and evaluate their potential as a more pathogenic form of biofilm, we analyzed the transcriptome of flow-induced microcolonies to identify core microcolony genes and TRs; and assessed the role of these candidate microcolony genes in epithelial cell adhesion, invasion and cell damage. *HWP1*, *ECE1*, *HYR1*, *PGA10*, *SAP5*, and *SOD5* were among the most highly expressed virulence factor genes among 20 core microcolony genes identified. We found that a third of core TRs for conventional biofilms were also identified in microcolony formation (Rob1, Ndt80, Efg1), but that Sfl1 and Sfl2 and other associated regulators of hyphal morphogenesis have a central and novel role in microcolony formation. Furthermore, Sfl1 and Sfl2 were core elements for *C*. *albicans* adhesion, invasion and damage of epithelial cells.

## Results

### Formation of microcolonies with flow conditions

**Fig 1:**
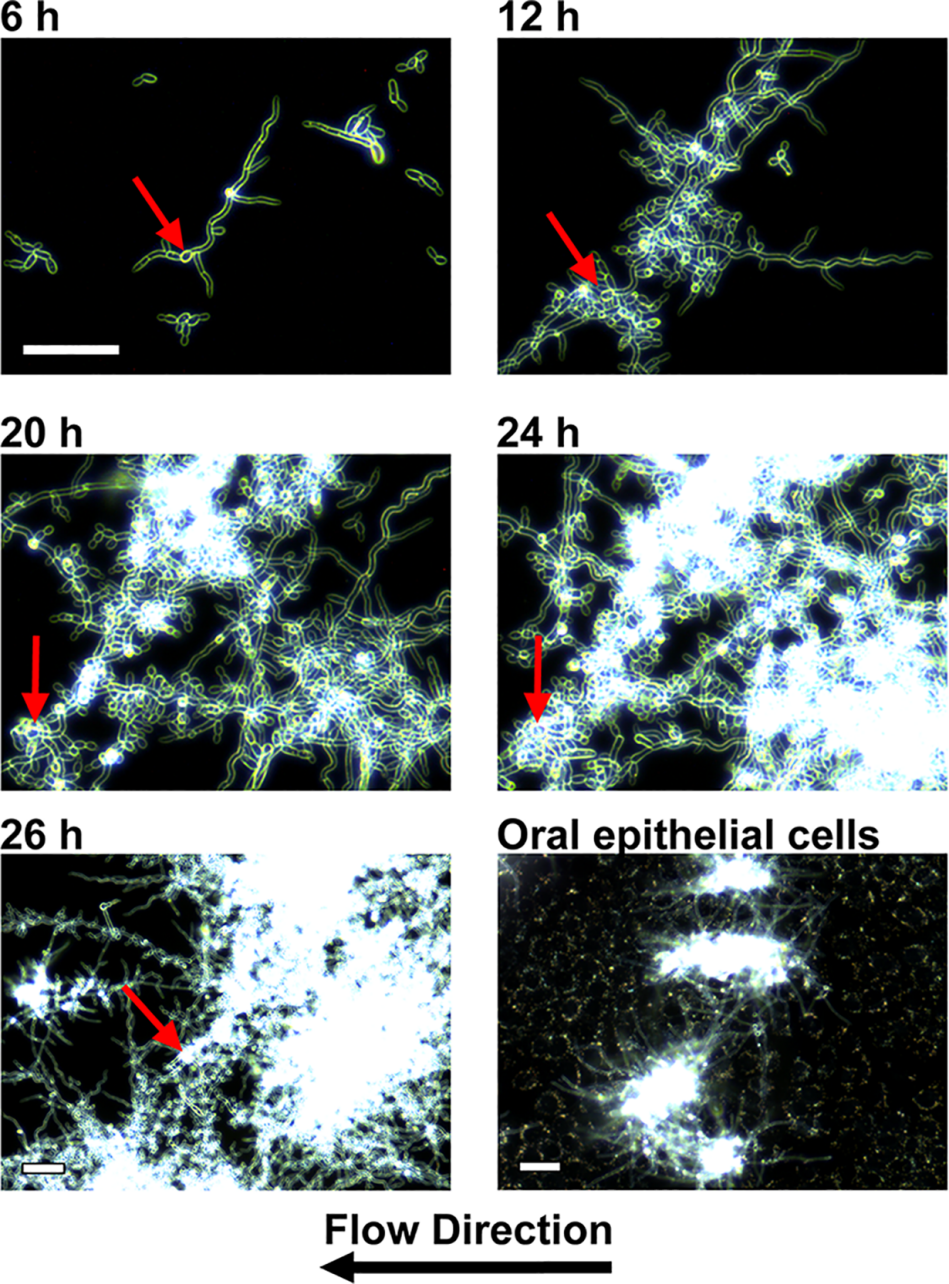
*C*. *albicans* forms microcolonies under continuous flow conditions. Wild-type *Candida albicans* cells were grown on plastic or epithelial monolayers under continuous flow at 37°C and imaged at indicated times using darkfield microscopy. Red arrows indicate the position of the originating mother cell in each image. Scale for upper four images is identical. Scale bars indicate 100 μm.

*C*. *albicans* WT cells (both CAI4 and SN250) that were grown under flow conditions in YPD medium at 37°C formed microcolonies in several stages (Fig 1 and S1 Video). Initially, yeast cells adhered to the surface and began to form multiple hyphal branches originating from one mother cell along the substrate surface over 6 h to form an early microcolony (Figs 1, 6 h; red arrow indicates originating mother cell). As hyphae continued to grow and branch over 12 h, they began to cross over one another and often developed into highly dense regions of the mid-phase microcolony. As hyphae continued to branch, they did not always orient away from the mother cell; instead many branched back towards existing clusters of overlapping hyphae or back towards the mother cell, contributing to the density of the mature microcolony (Fig 1, 20 h). Interestingly, the hyphal tip growth direction was not linear but typically sinusoidal in nature and unrelated to the direction of flow (see hyphae in Fig 1, 20 h at right). After 24 h, microcolonies became increasingly thick and merged with neighboring microcolonies, forming large structures that were easily observed with the unaided eye. The densest regions of mature (26 h) microcolonies were not in the vicinity of the mother cell, but rather were formed along the path of the sister hyphal branches. We consistently observed that additional germinated cells detached from the mother hyphal cell continually with flow over 26 h, suggesting a continuous role in microcolony dissemination. Additionally, a dramatic increase in lateral budding yeast and pseudohyphae were also observed along with continued dispersal and regrowth of new microcolonies nearby.

Formation of hyphal-based microcolonies under flow was unique, as this architecture was not observed when *C*. *albicans* cells were grown under flow at 22°C; and *C*. *albicans* cells grown without flow at 37°C on the flow system substrate formed conventional biofilms. We next cultured buccal epithelial cell monolayers (TR146) as a substrate within our flow system to confirm that microcolonies were also able to form on biotic as well as abiotic surfaces. Indeed, *C*. *albicans* cells formed distinct microcolonies within 6 h of infection upon epithelial cells (Fig 1, lower right panel), showing that microcolonies induced by flow at 37°C are formed both upon abiotic and epithelial substrates.

### Flow induces selective expression of genes involved in cell metabolism and virulence

**Fig 2:**
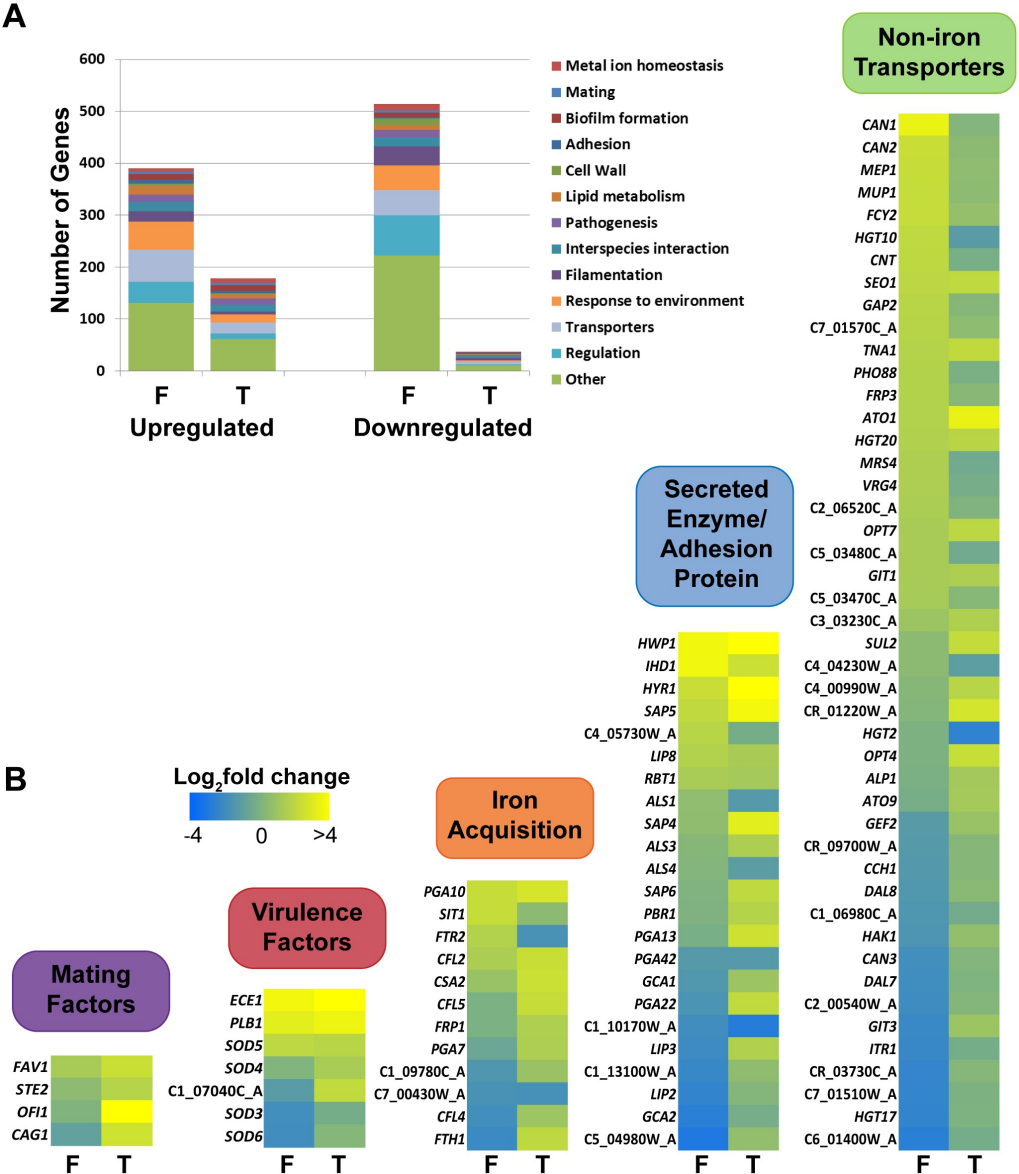
Flow induces more numbers of *C*. *albicans* genes having changes in expression level than does temperature. RNA-seq was performed on *Candida albicans* wild-type (WT) CAI4 cells grown under microcolony inducing conditions (37°C with flow) and compared to non-microcolony biofilms grown at 37°C without flow (**F**; 654 gene expression changes) or at 23°C with flow (**T**; 142 gene expression changes). (**A**) Gene ontology analysis of both RNA-seq datasets, generated from the *Candida* Genome Database. Genes may map to multiple ontological categories. (**B**) Differentially regulated genes from both RNA-seq datasets were hand annotated into five unique categories, with heatmaps of the expression data being shown. Genes with significantly different (Cuffdiff, P<0.05) changes in transcript levels in at least one dataset are shown.

Although 37°C temperature was permissive for *C*. *albicans* microcolony formation, we found that flow itself is a driving force initiating and propagating hyphal microcolony. To elucidate the transcriptome of microcolony formation under flow, gene expression profiles were obtained by RNA-seq and compared between microcolonies developed in flow at 37°C with biofilms grown in flow at 22°C (**T**); and microcolonies developed in flow at 37°C with static biofilms grown at 37°C (**F**) (Fig 2). When examining genes induced specifically by flow (**F**), we found 654 genes were differentially expressed (280 genes increased and 374 genes decreased). In contrast, only 117 genes were upregulated and 25 genes were downregulated specifically by temperature (**T**). Gene ontology (GO) analyses showed that a large subset of genes increased specifically by flow (**F**) were response to the environment, transporters, and cell regulation, as well as metabolic processes (RNA, DNA, lipid and vitamin metabolism, and cellular respiration) (Fig 2A), suggesting that nutrient availability in the flow system allows robust cellular metabolism and expression of genes involved in nutrient uptake. Temperature differences (**T**) induced higher proportions of differentially regulated genes related to metal ion homeostasis, biofilm formation, cell adhesion, pathogenesis, and interspecies interactions (Fig 2A). Although, there were minimal differences in the proportion of filamentation genes between the two datasets, the expression of 51 filamentation genes were changed specifically by flow compared with 13 genes with temperature, suggesting that flow may have an even greater impact on hyphae formation than does temperature.

As we were interested in estimating the growth and pathogenic capacity of flow-induced microcolonies, we further annotated our datasets using gene categories related to these processes, including metabolic transport (non-iron transporters), virulence, iron acquisition, extracellular enzymes/adhesion proteins, and mating (category placement was determined by canonical or predicted gene function) (Fig 2B). Several virulence factor genes (7 differentially regulated gene transcripts altered by flow, and transcripts altered by temperature) were enriched in our dataset including *ECE1* (also known as candidalysin) that has recently been shown to permeabilize the host membrane to increase nutrient acquisition [31], and several SOD proteins known to have critical roles in protecting *C*. *albicans* from host derived oxidative stress [32]. We found numerous iron acquisition genes (12 genes) that were differentially regulated. Most of the genes in this category were directly involved in acquiring iron, however, we also found numerous reductases that convert environmental iron to a form useable by the cell. A large group of adhesin genes were differentially regulated (14 genes) including genes encoding cell wall proteins Hwp1, Ihd1, and Hyr1. We also identified several proteases, lipases, and amylases (9 genes), as well as *SAP4*, *SAP5* and *SAP6* genes that play an important role in oral colonization and adhesion between *C*. *albicans* cells [2]. The largest group of genes (45 genes) differentially regulated in either dataset we classified as non-iron transport proteins. Most of these were transportation proteins related to metabolic processes, including amino acid/peptide transporters, carbon source transporters, nucleotide transporters, and other small metabolite transporters (mostly various co-enzyme factors). Most of these transcriptional changes (36 genes) were regulated by flow, while only 15 genes were temperature dependent, further illustrating the profound impact of flow on cellular metabolic processes. Lastly, we found that four genes involved in mating were significantly upregulated only in the temperature dataset. This aligns with previous reports showing that temperature affects expression of various mating factors [33] as well as impacting biofilm formation and virulence [34,35].

### The core microcolony response genes contribute to microcolony formation and size

Each dataset (flow and temperature) was then individually sorted by their fold change values and the ten genes with the highest fold change from each set were identified (Table 1).

**Table 1:** *C*. *albicans* most highly upregulated genes in microcolonies induced by flow and temperature. Ten genes with the highest fold change for Flow and Temperature datasets from RNA-seq analysis.

***C*. *albicans* Gene**	**Log**_**2**_ **(fold change)**
**Flow**	
*NUP*	5.49
*PGA45*	5.21
*GDP2*	5.05
*HWP1*	5.03
*ECE1*	5.00
*IHD1*	4.94
*CAN1*	4.70
*PLB1*	4.37
*AAH1*	3.89
*ROD1*	3.76
**Temperature**	
*ECE1*	9.09
*HWP1*	6.91
*OFI1*	6.45
*HYR1*	5.94
*SAP5*	5.05
*PLB1*	4.88
*ATO1*	4.66
C6_03600C_A	4.54
C7_03310W_A	4.51
C3_01540W_A	4.40

**Fig 3:**
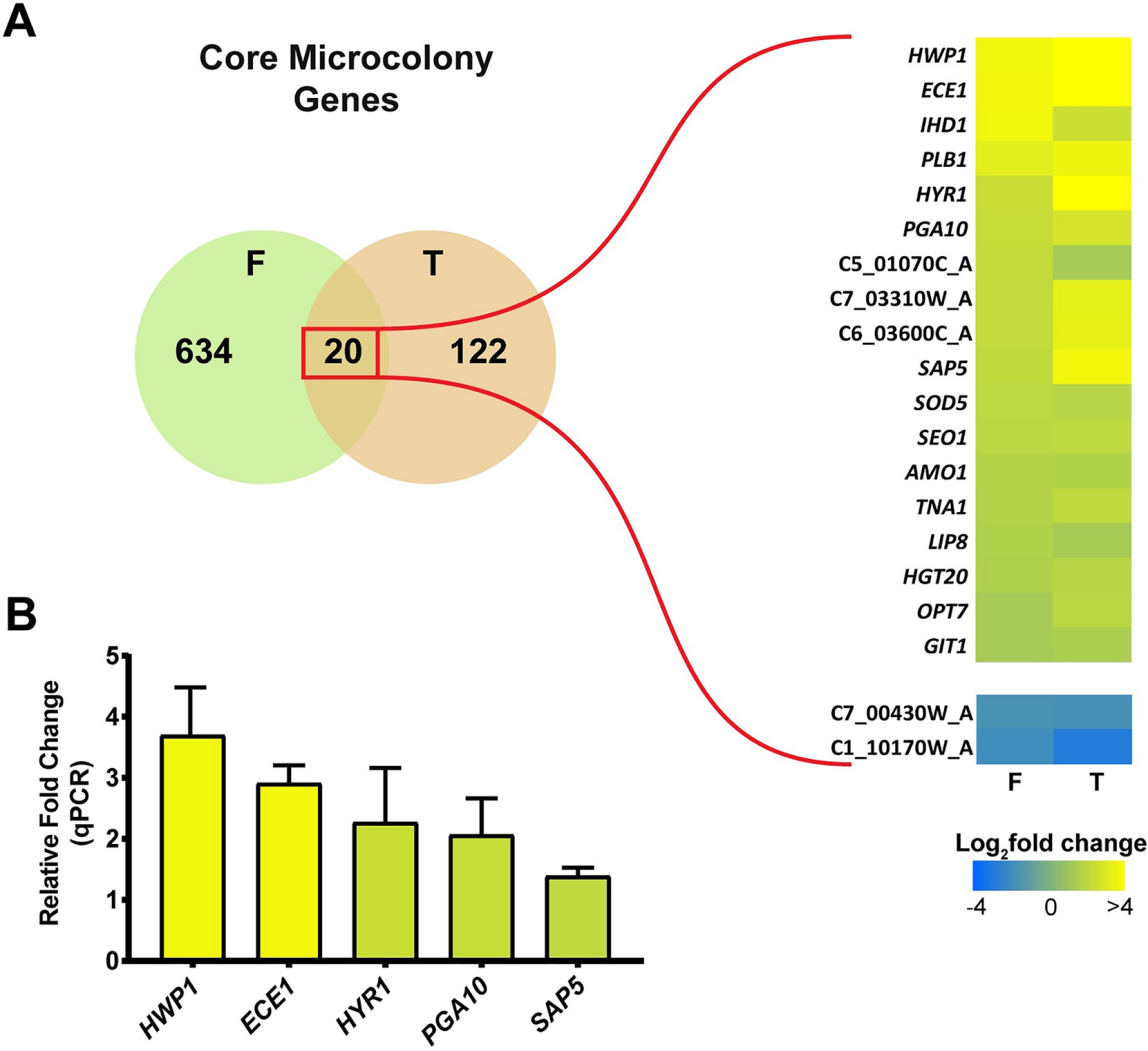
Twenty common genes are differentially regulated in microcolonies formed by both flow and temperature. (**A**) RNA-seq was performed on CAI4 biofilms grown under microcolony inducing conditions (37°C with flow) and compared to non-microcolony biofilms grown at 37°C without flow (**F**) or at 23°C with flow (**T**). Numbers in the Venn diagram indicate differentially regulated genes from each dataset with the genes in the overlap region also sharing the direction of regulation (increased or decreased in both datasets). A heatmap of the expression data of the 20 core microcolony genes is shown on the right. (**B**) Expression of the core microcolony genes *HWP1*, *ECE1*, *HYR1*, *PGA10* and *SAP5* was quantified by qRT-PCR on CAI4 microcolonies grown under flow and 37°C, and compared to CAI4 non-microcolony biofilms grown at 37°C without flow. Expression data was first normalized to control actin prior to comparison between samples. The results represent the averages from triplicate samples from two independent experiments. The error bars indicate standard deviations.

Interestingly, many of the highest up-regulated genes including *ECE1* and *HWP1* were common to both sets. Therefore, we merged genes from those differentially expressed genes common to both datasets (634 **F** and 122 **T**) into a final set of 20 core microcolony genes that were differentially regulated in the same direction (18 up-regulated and 2 down-regulated in both datasets) (Fig 3A). We further validated this data set using RT-qPCR by determining expression level of core microcolony genes (*HWP1*, *ECE1*, *HYR1*, *PGA10* and *SAP5*) in flow-induced microcolonies. RT-qPCR analyses confirmed that *HWP1* was highly up-regulated in flow induced microcolony formation (by 3.6 fold), as well as *ECE1* (by 2.9 fold), *HYR1* (by 2.2 fold) *PGA10* (by 2.0 fold) and *SAP5* (by 1.39 fold) compared with these genes in static biofilms without CO_2_ (Fig 3B). Notably, the majority of core up-regulated genes are involved in cell adhesion, biofilm formation, pathogenesis, nutrient transport, or oxidation-reduction processes.

**Fig 4:**
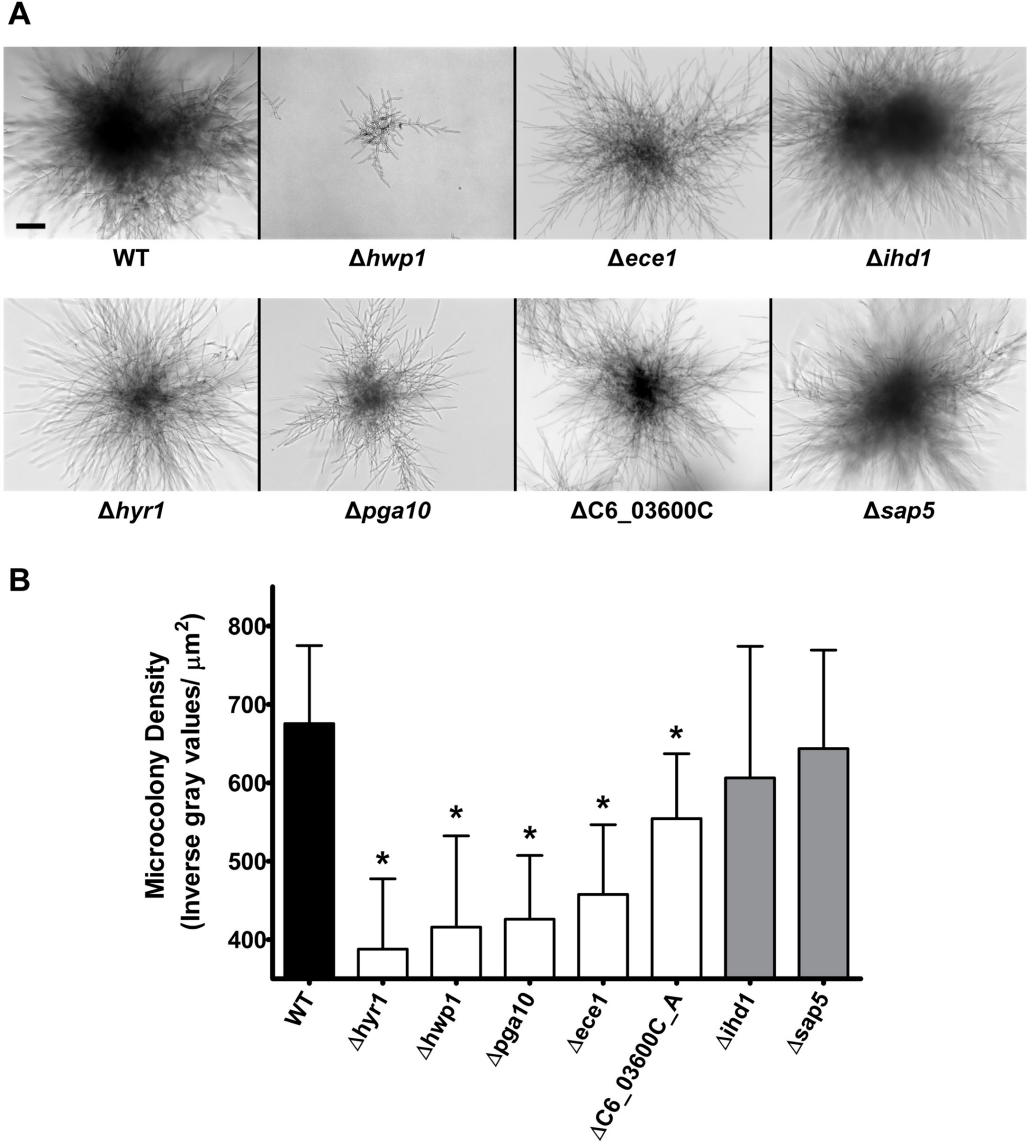
Five core microcolony genes are essential for microcolony formation. (**A**) Homozygous knockout mutants and wild type CAI4 cells were grown under static microcolony inducing conditions (RPMI with 5% CO_2_) for 20 h, and imaged using brightfield microscopy. Scale bar indicates 100 μm. (**B**) Microcolony density per square micron was evaluated for each strain using ImageJ. Data are means ± SD of n≥3 experiments, with * indicating significance by a post-hoc Tukey’s test at *p* < 0.05 as compared to WT.

To validate the role of these core microcolony genes for microcolony formation (as well as growth and hyphal defects), we evaluated *C*. *albicans* gene deletion strains for microcolony formation using RPMI and 5% CO_2_ (Fig 4), since most of these mutants had reduced adhesion and were not able to form biofilms or microcolonies in our flow apparatus. None of the gene deletion mutants had abnormal filamentation in conventional assays; however several of these mutants were significantly defective in microcolony formation (Fig 4, and S1 Fig). Consistent with their ranking, the highest up-regulated core genes (Fig 3) also had the largest defects in microcolony formation using core gene deletion mutants (Δ*hwp1*, Δ*ece1*, Δ*hyr1*, and Δ*pga10*). *C*. *albicans* Δ*hwp1* showed the strongest defect having significantly smaller microcolonies and hyphal-branch lengths. We also noticed that the hyphae of the Δ*hyr1* cells appeared to branch significantly less frequently leading to more outward growth with fewer overlapping regions; suggesting a novel role for *HYR1* in either cell wall remodeling or signaling related to hyphal branching. *C*. *albicans* Δ*ece1*, Δ*hyr1*, ΔC6_03600C, Δ*pga10* microcolonies (Fig 4), as well as Δ*opt7* (S1 Fig), although relatively similar in diameter to WT, displayed a significant decrease in microcolony density. The remaining lower ranked core microcolony genes including Δ*ihd1*, Δ*sap5*, (Fig 4) as well as Δ*lip8* (S1 Fig) showed no significant differences compared to WT colonies. Thus, at least six core microcolony genes, *HWP1*, *ECE1*, *HYR1*, C6_03600C, *OPT7* and *PGA10* are important for establishing the size and density of a microcolony, while other core genes may have ancillary roles.

### Predicted transcription factors play key roles in microcolony development

To gain a better understanding of the regulatory elements related to flow-induced microcolony formation, candidate transcriptional regulators (TRs) were identified using the PathoYeastract database to predict TRs of the 20 core microcolony genes. We discovered 10 TRs that regulated 50% or more of the core microcolony genes (Table 2; S2 Fig). Three TRs (Rob1, Ndt80, and Efg1) are known to play key roles in static *in vitro* biofilm formation; while seven other TRs that previously have not been known to affect biofilm formation were identified (Mcm1, Ndt80, Tye71, Rca1, Sfl1, Sfl2, and Fkh2). Furthermore, all of these TRs have been shown to play a role in hyphal morphogenesis, underscoring the importance of hyphae in microcolony formation.

**Fig 5:**
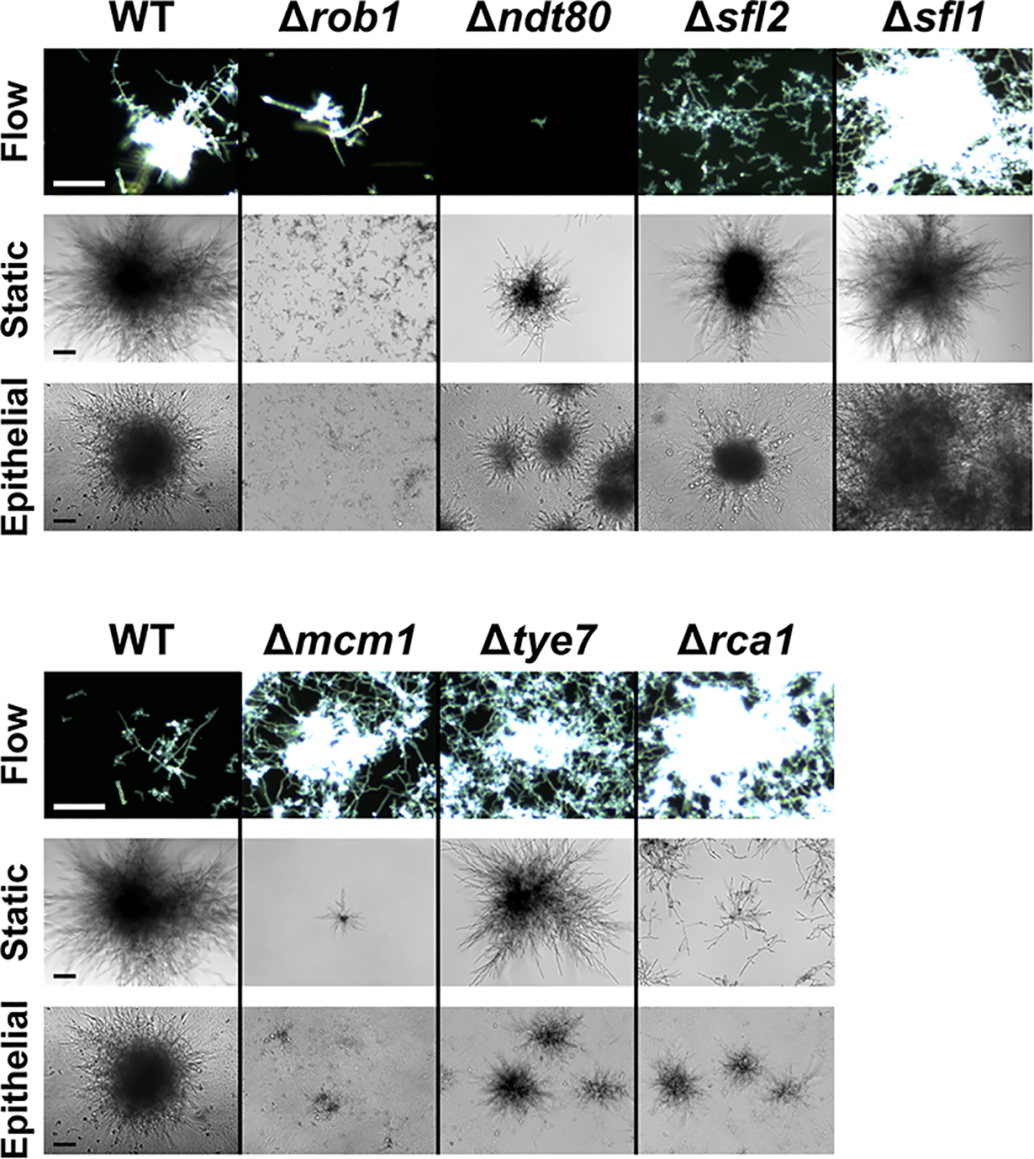
*Candida albicans* transcriptional regulator knockouts Δ*rob1*, Δ*ndt80*, and Δ*sfl1* showed microcolony formation defects. *Candida albicans* wild-type (WT) cells and seven transcriptional regulator knockouts were evaluated for their microcolony formation using flow assay, and static plate assays (RPMI and 5% CO_2_) on both plastic and epithelium. Flow microcolonies were imaged using time-lapse microscopy. Flow sample images show results at 18 h (WT [top], Δ*rob1*, Δ*ndt80*, and Δ*sfl1*), 8 h (WT [bottom], Δ*mcm1*, Δ*tye7*, and Δ*rca1*), and 24 h (Δ*sfl2*). Static microcolonies were grown for 24 h prior to imaging. Microcolonies that showed consistent differences in both static and flow conditions when compared to WT are on the top row, with most showing reduced microcolony formation (except Δ*sfl1* that had larger microcolonies). The three remaining mutants (bottom row) had inconsistent results between static and flow conditions, with flow microcolonies being more dense and static microcolonies being reduced in size. Scale bars indicate 100 μm.

We compared the ability of these seven *C*. *albicans* TR deletion mutants with WT cells to assess their contribution to microcolony formation and density in both flow and static conditions (abiotic and epithelial monolayers of TR146 cells) (Fig 5 and S3 Fig). We could not assess the contribution of three TRs in flow: *C*. *albicans Efg1* mutants failed to form microcolonies due to filamentation defects (S2 Video); *C*. *albicans* Δ*nrg1* cells had extensive flocculation; and *C*. *albicans* Δ*fkh2* cells had significant growth defects. Of the remaining seven TRs, all were capable of filamentation and exhibited microcolony formation in the flow assay.

Among the seven identified TRs, Rob1, Ndt80, Sfl1 and Sfl2 were found to substantially affect microcolony formation under all conditions (flow, static, and epithelial monolayers); while three (Mcm1, Tye7, Rca1) had inconsistent affects. *C*. *albicans* Δ*rob1* cells were able to filament and formed very small microcolonies when grown with flow, (Fig 5, S3 Video), and were completely defective in microcolony formation in static conditions or on epithelial cells. Microcolony formation in *C*. *albicans* Δ*ndt80* cells in static conditions was reduced compared to WT cells although they produced typical filamented colonies; however Δ*ndt80* cells were completely deficient in filamentation with flow, instead behaving more like Δ*efg1* with no hyphal formation and generally low adhesion (Fig 5, S4 Video). *C*. *albicans SFL1* and *SFL2* are antagonistic hyphal morphogenesis regulators, with *SFL2* promoting hyphal growth, and *SFL1* being a negative regulator of hyphal morphogenesis [29]. As expected, *C*. *albicans* Δ*sfl1* formed very large microcolonies by 18 h compared to WT cells (Fig 5 and S5 Video), while Δ*sfl2* cells had less robust filamentation and formed very small microcolonies (Fig 5 and S6 Video). This phenotype was consistent (Δ*sfl1* larger and Δ*sfl2* smaller microcolonies) in both abiotic and epithelial static conditions. Complementation of *SFL1*, *SFL2*, and *ROB1* into their respective deletion mutants restored microcolony density to WT levels under static growth conditions.

**Table 2:** Ten predicted microcolony transcriptional regulators. Twenty core *C*. *albicans* microcolony genes were analyzed using the PathoYeastract database to predict transcription factors that regulated at least half of the core microcolony genes.

	Percent genes regulated	Genes regulated
***C*. *albicans* TR**		
**Rob1**	**70%**	*C1_10170W_A*, *HYR1*, *SOD5*, *GIT1*, *ECE1*, *HWP1*, *C5_01070C_A*, *TNA1*, *SAP5*, *C6_03600C_A*, *IHD1*, *C7_00430W_A*, *LIP8*, *C7_03310W_A*
**Mcm1**	**70%**	*C1_10170W_A*, *HYR1*, *SOD5*, *HGT20*, *AMO1*, *GIT1*, *ECE1*, *HWP1*, *C5_01070C_A*, *SAP5*, *C6_03600C_A*, *IHD1*, *C7_00430W_A*, *SEO1*
**Nrg1**	**65%**	*C1_10170W_A*, *HYR1*, *SOD5*, *HGT20*, *OPT7*, *PGA10*, *ECE1*, *HWP1*, *C5_01070C_A*, *PLB1*, *SAP5*, *IHD1*, *C7_03310W_A*
**Efg1**	**65%**	*HYR1*, *SOD5*, *GIT1*, *PGA10*, *ECE1*, *HWP1*, *TNA1*, *SAP5*, *C6_03600C_A*, *IHD1*, *C7_00430W_A*, *LIP8*, *C7_03310W_A*
**Ndt80**	**60%**	*HYR1*, *SOD5*, *AMO1*, *GIT1*, *OPT7*, *ECE1*, *HWP1*, *C5_01070C_A*, *SAP5*, *C6_03600C_A*, *IHD1*, *C7_00430W_A*
**Tye7**	**60%**	*HYR1*, *SOD5*, *AMO1*, *GIT1*, *PGA10*, *ECE1*, *HWP1*, *C5_01070C_A*, *TNA1*, *IHD1*, *C7_00430W_A*, *SEO1*
**Rca1**	**55%**	*HYR1*, *HGT20*, *AMO1*, *GIT1*, *OPT7*, *ECE1*, *HWP1*, *TNA1*, *IHD1*, *C7_03310W_A*, *SEO1*
**Sfl2**	**55%**	*HYR1*, *AMO1*, *GIT1*, *OPT7*, *PGA10*, *ECE1*, *HWP1*, *TNA1*, *PLB1*, *SAP5*, *IHD1*
**Fkh2**	**50%**	*HYR1*, *SOD5*, *PGA10*, *ECE1*, *HWP1*, *C5_01070C_A*, *SAP5*, *IHD1*, *C7_03310W_A*, *SEO1*
**Sfl1**	**50%**	*HYR1*, *SOD5*, *AMO1*, *ECE1*, *HWP1*, *C5_01070C_A*, *SAP5*, *C6_03600C_A*, *IHD1*, *C7_00430W_A*

Other *C*. *albicans* knockouts including Δ*mcm1*, Δ*tye7* and Δ*rca1* all were defective in microcolony formation when grown statically (both abiotic and upon epithelium); however, this group of TR knockouts showed more robust microcolony formation under flow than WT cells (Fig 5 lower panel, S7–S9 Videos). These knockouts had high adhesion to the substrate and formed fast-growing dense microcolonies with flow. These differences in microcolony phenotypes may be due to environmental differences (oxygen or CO_2_ levels and media) or a mechanosensory response with flow. In either case, our data suggest that *RCA1*, *TYE7*, and *MCM1* are unlikely to be a part of the central regulatory mechanism for microcolony formation.

**Fig 6:**
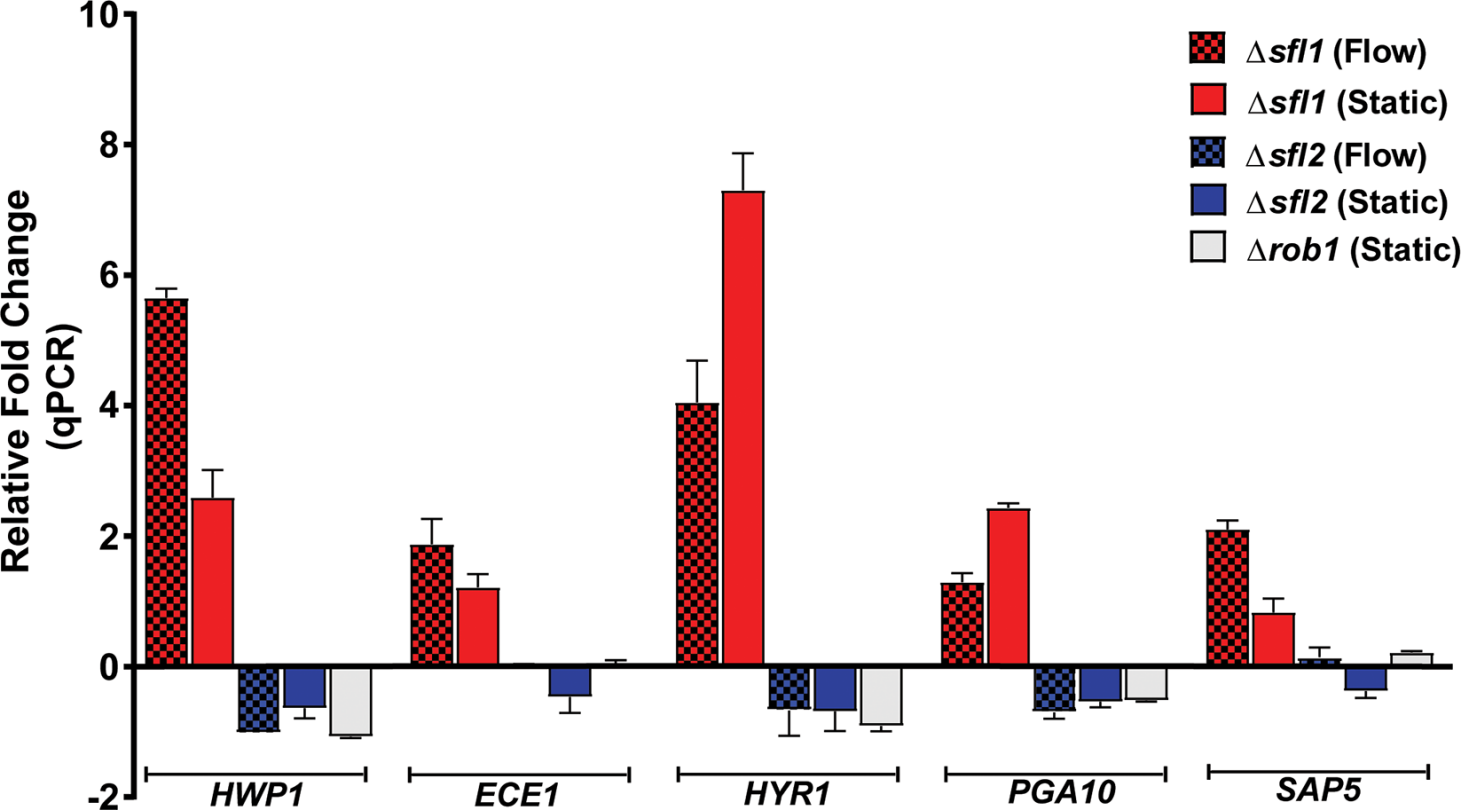
Quantitative real-time RT-PCR analysis of core microcolony genes under flow and static conditions. Expression of the core microcolony genes *HWP1*, *ECE1*, *HYR1*, *PGA10* and *SAP5* was quantified by qRT-PCR experiments in Δ*sfl1*, Δ*sfl2* and Δ*rob1* strains grown under flow and static conditions at 37°C. Expression of the core microcolony genes were highly up-regulated in Δ*sfl1* cells (flow indicated by red hatched bars, static by red solid bars), and down-regulated in both Δ*sfl2* (flow indicated by blue hatched bars, static by blue solid bars) and Δ*rob1* (static white bars) microcolonies. Bars in each graph indicate relative fold changes in RNA expression of each sample as compared to control actin. The results represent the averages from triplicate samples from two independent experiments. The error bars indicate standard deviations.

Since core microcolony genes were highly expressed in flow-induced microcolonies, and their deletion resulted in defective microcolony phenotype, we predicted that the microcolony TR complex would regulate expression levels of core microcolony genes. We expected that *SFL1* and *SFL2* TRs (and perhaps *ROB1*) would regulate the expression of core microcolony genes (*HWP1*, *ECE1*, *HYR1*, *PGA10* and *SAP5*) in both flow and static microcolonies. Therefore, we compared expression levels of these genes from flow and static microcolonies of Δ*sfl1*, Δ*sfl2* and Δ*rob1* strains with WT microcolonies using RT-qPCR (Fig 6). All five core microcolony genes were up-regulated in the Δ*sfl1* strain which forms very large microcolonies in both flow or static conditions; while these same genes were down-regulated in the Δ*sfl2* strain which is defective in microcolony formation. As expected, the Δ*rob1* strain, which had severe defects in microcolony formation (only static microcolonies provided sufficient RNA for analyses), also had reduced expression of all five core microcolony genes. Among the five genes examined, *HWP1* and *HYR1* genes were most highly modulated by *SFL1*, *SFL2*, and *ROB1*; however these changes were independent of hyphal formation since expression levels of hyphal specific cyclin *HGC1* were unchanged in these TR deletion strains.

### The microcolony TR network participates in adhesion, invasion, and cytotoxicity with epithelial cells

*In vitro* infection process on oral epithelial cells has been categorized in three phases: Adhesion phase where >90% candida cells attaches to epithelial cells within 3h., Invasion phase (3-12h) with >75% invading hyphae within 4h, and lastly robust tissue damage (12-24h). We followed similar time period to study role of microcolony TR network in adhesion, invasion and epithelial cytotoxicity.

**Fig 7:**
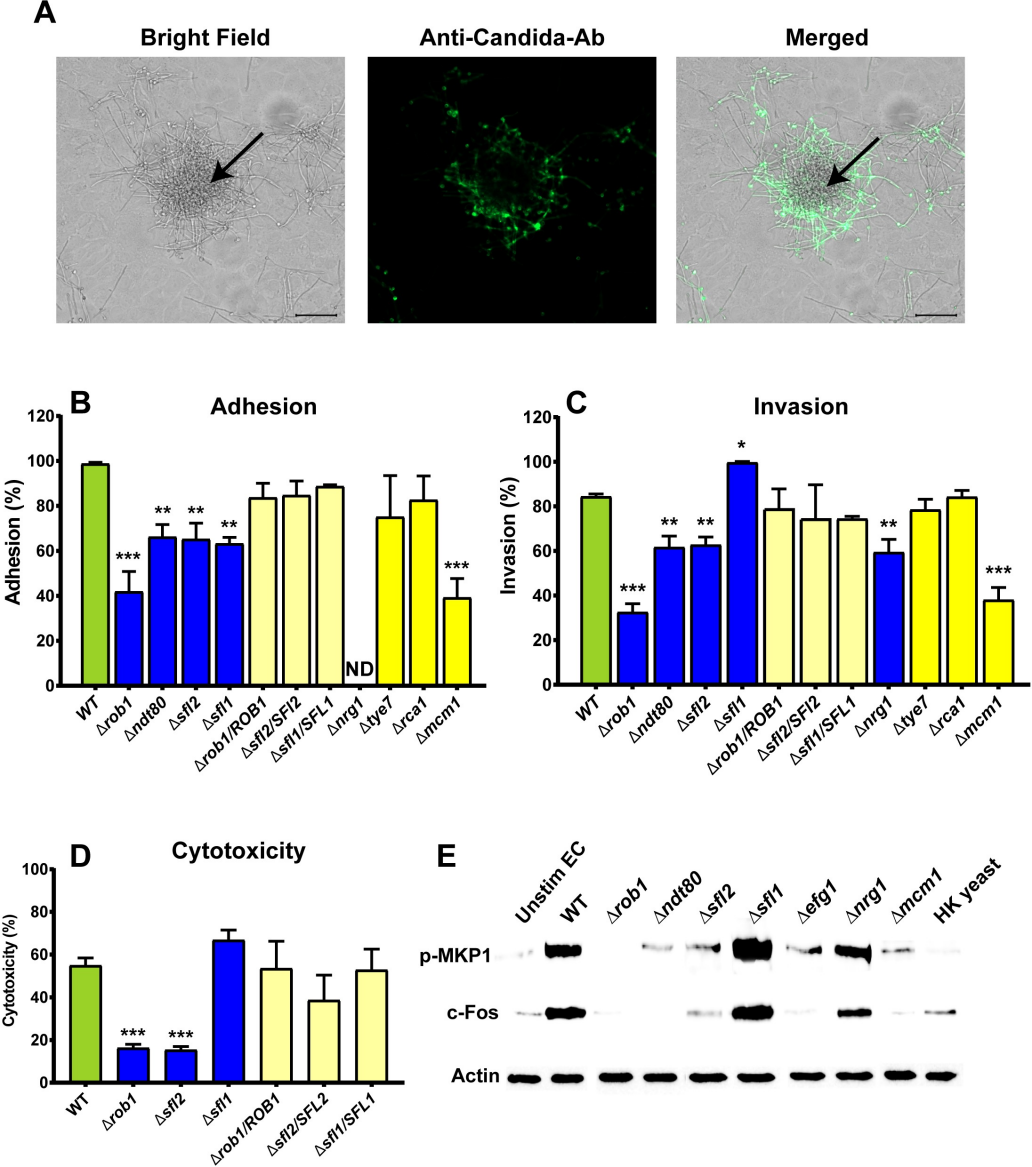
*Candida albicans* transcriptional regulators Rob1, Ndt80, Sfl1 and Sfl2 are involved in adhesion, invasion and damage of epithelial monolayers. (**A**) Wild-type (WT) *Candida albicans* CAI4 cells (1 × 10^5^) were added to TR146 buccal epithelial squamous cell monolayers in serum-free DMEM/F-12 medium and incubated for 12 h. Non-adherent *C*. *albicans* cells were removed by washing, and adherent cells fixed with 4% formaldehyde. Epithelial cells were permeabilized and adherent *Candida* cells were stained with anti-*Candida* antibody and Alexa Fluor 488. *C*. *albicans* cells invading epithelium were not stained (center of microcolony with black arrow), while surface adhered non-invading cells were stained with anti-*Candida* antibody (green). Scale bar indicates 20 μm. (**B** & **C**) *C*. *albicans* knockouts of eight transcriptional regulators were quantitated for adhesion (90 min incubation) and invasion (4.5 h) on epithelial monolayers and compared to WT cells. Asterisks indicate statistically significant differences compared to WT cells, * p<0.05, ** p<0.01, *** p<0.001. ND: No data. (**D**) TR146 cells were infected with WT cells and transcription regulator deletion strains for 24 h, and cell damage was measured by LDH release as cytotoxicity (%). There was no significant change in LDH release in TR146 cells infected with the Δ*sfl1* and complemented strains of *SFL1*, *SFL2* and *ROB1* compared to cells infected with WT. Both the Δ*sfl2* and Δ*rob1* strains induced significantly less cell damage (***, P < 0.001). The results represent the averages from triplicate samples from two independent experiments. The error bars indicate standard deviations. (**E**) TR146 epithelial cells were infected with each *Candida* strain at MOI (1:10) for 3 h. After infection, epithelial cells were lysed, left on ice for 30 min, then isolated and immunoblotted with Phospo-MKP1 [S359] and c-Fos using rabbit monoclonal antibodies. Actin was used as a loading control.

To confirm that microcolonies formed on epithelial cells (12h post infection) possess invasive properties, differential staining using a *Candida*-specific antibody was done to detect microcolony invasion at 12h. Indeed, large regions of the microcolony were found to invade underlying epithelial cells (Fig 7A, arrow), while other regions of the microcolony remained attached to the surface.

To determine whether the same TRs that effect microcolony formation also influenced invasion, we further assessed the ability of microcolony defective *C*. *albicans* TR knockouts to adhere and to invade oral epithelial cells (Fig 7B and 7C and Table 3). Once contacted with epithelial monolayers, *C*.*albicans* WT cells showed >90% adhesion within 2h of infection and >80% invasion within 4.5h of infection. We found that four microcolony deficient strains (*Δrob1*, *Δndt80*, *Δsfl1* and *Δsfl2*) were also significantly defective in adhesion with epithelial cells and these defects were reversed in complemented strains (Fig 7B).

**Table 3:** Knockout of five transcriptional regulators (TR) and two core microcolony (MC) genes result in defects in microcolony formation and function. *C*. *albicans* deletion mutants were grown on oral epithelial cells (OEC); and microcolony formation, adhesion and invasion were measured. Significant (*p* < 0.05) differences in microcolony density, number of adhered cells, or percentage of cells actively invading were determined using one-way ANOVA followed by a post-hoc Tukey’s t-test (microcolony formation) or Bonferroni (adhesion and invasion). Strains highlighted in grey were defective in all three categories. ND: not determined.

	Microcolony Formation	OEC Adhesion	OEC Invasion
***C*. *albicans* TR**			
***Δrob1***	**Defective**	**Defective**	**Defective**
***Δndt80***	**Defective**	**Defective**	**Defective**
***Δsfl1***	**Increased**	**Defective**	**Increased**
***Δsfl2***	**Defective**	**Defective**	**Defective**
***Δmcm1***	**Defective**	**Defective**	**Defective**
***Δtye7***	**Defective**	No defect	No defect
***Δrca1***	**Defective**	No defect	No defect
***Δnrg1***	**Defective**	ND	**Defective**
**Core MC Genes**			
***Δhwp1***	**Defective**	**Defective**	**Defective**
***Δece1***	**Defective**	No defect	No defect
*Δihd1*	No Defect	No defect	No defect
***Δhyr1***	**Defective**	No defect	No defect
***Δpga10***	**Defective**	**Defective**	**Defective**
*Δ*C6_03600C_A	No Defect	No Defect	No Defect
*Δsap5*	No Defect	**Defective**	**Defective**
*Δlip8*	No Defect	No defect	No defect

As expected for loss of its repressor function, *Δsfl1* cells had significantly increased epithelial invasion compared to WT cells; while *Δrob1*, *Δndt80* and *Δsfl2* cells had reduced invasion (Fig 7C). Complementation of these gene knockouts (*Δ*sfl1*/SFL1*, *Δrob1/ROB1* and *Δsfl2/SFL2*) in all cases restored the WT phenotype. Due to its hyper-filamentous nature, we could not determine adhesion for Δ*nrg1*, although these cells were significantly defective for host invasion. Cells with deletion of *TYE7* and *RCA1* did not show any changes in adhesion and invasion on host cells. Although *Δmcm1* cells had no defect in microcolony formation in flow, these cells showed significantly reduced adhesion and invasion on epithelial monolayers, suggesting its role in microcolony formation specifically under static conditions.

We further tested other core microcolony response genes for their role in adhesion and invasion (S4 Fig) and found *Δhwp1* and *Δpga10* had significantly reduced adhesion (19% and 29%) and invasion (63% and 49%) to epithelial cells while *Δece1* and *Δhyr1* cells did not have significant changes in adhesion or invasion, consistent with previous reports [31,36,37]. Interestingly, the secreted aspartyl protease mutant *Δsap5* was defective for adhesion (50%) and invasion (61%) even though it was not defective in microcolony formation. Together these results demonstrate that Hwp1 and Pga10 are important proteins involved microcolony formation as well as initial adhesion and invasion; and that Ndt80, Rob1, Sfl1, and Sfl2 all play central roles in not only the development and architecture of microcolonies, but also in their pathogenic properties.

We further investigated if microcolony defective TRs were also defective in their ability to elicit epithelial cell damage (Fig 7D). Culture supernatant was examined after 24 h of infection for levels of lactate dehydrogenase (LDH), a measure for host cell damage. We found that the *Δsfl2* (15%) and *Δrob1* (16%) strains induced significantly less cytotoxicity than the wild type strain (52%), and *Δsfl1* cells induced higher cell damage (66%) although *Δsfl1* did not reach statistical significance. Complemented strains *Δ*sfl1*/SFL1* (53%), *Δrob1/ROB1* (54%) and *Δsfl2/SFL2* (38%) had statistically equal cytotoxicity as WT cells.

Oral epithelial cells concurrently sense and respond to *C*. *albicans* hyphae by activation of the MKP1/c-Fos pathway during initial adhesion and invasion, leading to a cytokine response [30]. We expected that the microcolony TR network, which is required for epithelial adhesion and invasion, also mediates activation of the MAPK-based MKP1/c-Fos signaling pathway. To determine the role of microcolony formation in the activation of epithelial cells, we evaluated microcolonies of TR mutants for MKP1 phosphorylation and c-Fos activation (Fig 7E). Western blot analysis of TR146 oral epithelial cells demonstrated that *Δrob1*, *Δndt80* and *Δmcm1* strains did not induce p-MKP1 and c-Fos activation; similar to yeast locked Δ*efg1* and heat killed yeast cells. Furthermore, deletion of *SFL1* resulted in hyper-filamentation and increased levels of p-MKP1 and c-Fos in epithelial cells compared to WT, while in contrast the hyper-filamentous *Δnrg1* behaved similar to WT; showing that Sfl1 virulence is not due solely to its hyperfilamentous phenotype even though it may reduce its adhesion to surface. As expected, *Δsfl2* cells had reduced adhesion and invasion of epithelial cells compared to WT.

## Discussion

Microcolony formation is excellent example of the genomic and phenotypic plasticity of *C*. *albicans* that enable this pathogen to colonize a wide range of surfaces. As a dense mass of hyphae, microcolony formation enables *C*. *albicans* to strongly adhere, invade and forage for nutrients as a community of cells. We show for the first time that *C*. *albicans* microcolonies express a specific set of virulence and metabolic genes required to colonize surfaces in response to environmental cues. These environmental signals, including flow, initiate a transcriptional regulon of core microcolony genes that are differentially expressed depending on host environment. Involvement of multiple *C*. *albicans* TRs within the microcolony transcriptional network might be a strategy for adaptation to diverse host environments such as the oral cavity or gut.

The yeast-to-hyphae transition is induced by a wide variety of conditions such as temperature, nutrient availability, CO_2_ and certain media; however, we discovered that flow conditions are additional stimuli for hyphal production and microcolony formation. Our transcriptional analyses of flow-induced microcolonies identified 4-fold more changes in gene expression induced by flow as compared to a change in temperature (654 and 142 respectively); and these up-regulated genes were enriched in metabolic pathways. This is likely due to the flow system bringing fresh nutrients and oxygen to the developing biofilm, compared with static biofilm conditions in which the media is slowly depleted of nutrients [38]. This flow system is expected to be a good model of biofilm growth in the oral environment in which saliva is constantly replenished while providing cells with fresh nutrients; but is likely not representative of deep tissue infection. We found that human saliva was not amenable for use in this flow system as it clogged filters. Alternatively, *C*. *albicans* microcolonies were grown on saliva-coated flow chamber slides, rather than saliva-mimetic media that does not closely replicate the fungal or bacterial growth characteristics of saliva [39,40]. However, saliva coating did not result in any phenotypic differences compared to uncoated slides, suggesting that yeast growth media is representative of saliva at least for initial adhesion and growth of microcolonies.

We identified a set of core microcolony genes that included well known hyphal-specific genes (*HWP1*, *ECE1*, *IHD1*, *HYR1*, and *SOD5*) [34], as well as genes having roles in cell adhesion (*HWP1*, *IHD1*, and *HYR1*) [36], or nutrient acquisition from either the host or the environment, (*ECE1*, candidalysin is a protein that permeabilizes the membranes of epithelial cells; *SAP5* is a secreted protease, and *PGA10* required for iron acquisition) [31,41]; and metabolism (*TNA1*, *LIP8*, *AMO1*, *HGT20*, and *GIT1*) [42–45]. However, these identified core microcolony genes were not identical with the set of known hyphal specific genes; since several genes known to be highly up-regulated during hyphae formation such as *ALS3*, *ALS1*, *HGC1* and *RBT4* [46–48] were not increased in flow-induced microcolonies (Fig 3 and S1 Table). Interestingly, knockout strains of many of these core microcolony genes could not be tested in our flow assay, since the majority had substantial adhesion defects [36], further underscoring the role of cell adhesion for flow-induced microcolony formation. Using a static plate assay, we found several knock-out mutants (Δ*hwp1*, Δ*ece1*, Δ*hyr1*, and Δ*pga10*) had significant defects in microcolony formation, in some cases consistent with their known roles as adhesion proteins whose expression is induced during hyphal growth (Hwp1 and Hyr1) [36,49]. We were surprised to find Δ*ece1* cells had significantly reduced microcolony formation, as Ece1 previously was shown to permeabilize host membranes, a function that is unnecessary in our assay. Despite having reduced microcolony density, Δ*ece1* microcolonies grew to equivalent diameters as WT, showing that hyphal elongation and branching were unaltered in these mutants. An additional function for *ECE1* in microcolony formation is suggested by experiments in which *ECE1* over-expression restored biofilm formation in a Δ*bcr1* knockout strain, presumably through an adhesion based mechanism [49]. Similarly, although the GPI-anchored membrane protein Pga10 is known to be involved in iron acquisition and biofilm growth, our data suggest other roles in microcolony formation, perhaps also through surface adhesion. Together, these mutant strains suggest that initial adhesion and maintenance is an important factor for microcolony growth and development.

**Fig 8:**
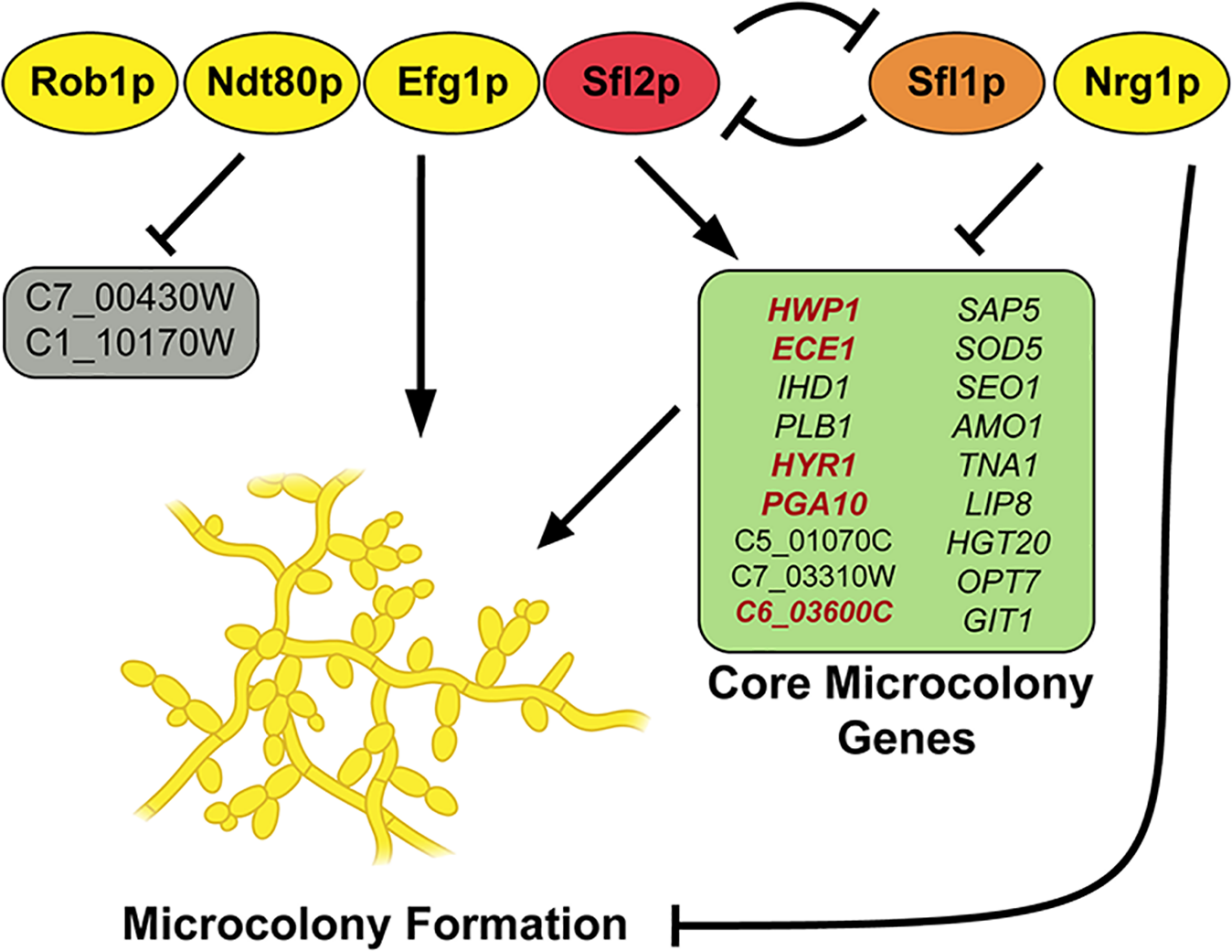
Model of the regulatory elements of *Candida albicans* microcolony formation. Six core microcolony transcriptional regulators (four positive [left], two negative [right]) are involved in microcolony formation, and regulation of core microcolony genes. *SFL1* (orange) and *SFL2* (red) have antagonistic roles, *SFL2* is active at higher temperatures and inhibits *SFL1* and *NRG1*, while *SFL1* and *NRG1* are active at lower temperatures. In microcolony forming conditions, the four positive microcolony transcriptional regulators (Rob1p, Ndt80p, Efg1p, and Sfl2p) work together to regulate the core microcolony genes (arrows indicate upregulation/promotion and blunt ends indicate downregulation/inhibition), and promote the formation and function (adhesion and invasion) of microcolonies. Core microcolony genes (in red) influence the size or density of microcolonies.

There have been numerous studies on the genetic network of the classical *in vitro* model for biofilm formation. Fox *et al*. [12] recently expanded on the work of Nobile *et al*., [11] that together characterize a network of nine TFs comprised of Brg1, Rob1, Ndt80, Tec1, Efg1, Bcr1, Gal4, Flo8, and Rfx2 that regulate biofilm formation. All of these, with the exception of Gal4 and Rfx2, are positive regulators of biofilm formation, such that their knockout shows drastic reduction in biofilm formation. These seven positive regulators were also shown to play important roles in hyphae formation, suggesting a key role of hyphal development in classical biofilm formation. Our work identified 10 TFs regulating ≥ 50% of flow-induced core microcolony genes, which included three of the previously identified biofilm formation factors (Rob1, Ndt80, and Efg1); while several others (Brg1, Tec1, Bcr1, and Flo8) regulated only 25–40% of core microcolony genes (S2 Table). Instead, our profile of TF regulators appears much more similar to the Sfl1/Sfl2 regulatory network recently described as the master on-off switch to coordinate *C*. *albicans* morphogenesis and virulence [29]. This elegant work showed that Sfl1 and Sfl2 respectively act as repressor and activator transcriptional modules that physically associate with Efg1, Rob1 and Ndt80 to affect filamentation. Our results further showed that the TR pair Sfl1 and Sfl2 had opposing and central effects for microcolony formation, as well as adhesion and invasion of oral epithelial cells (Fig 7B and 7C). Furthermore, Efg1, Rob1 and Nrg1 were also critical for microcolony development as Δ*efg1* and Δ*rob1* were incapable of forming microcolonies, while the Δ*nrg1* strain was hyperfilamentous. Additionally, we found that epithelial cells exhibited a reduced immune response to Δ*rob1*, Δ*ndt80* and Δ*sfl2* microcolonies (Fig 7E). Thus, we suggest that the Sfl1/Sfl2 regulatory network not only controls temperature dependent filamentation, but also is the dominant mechanism governing microcolony formation and function (Fig 8).

Interestingly, we identified three TRs (Mcm1, Tye7, and Rca1) that are not part of the Sfl1/2 regulatory network whose knockouts had smaller CO_2_-induced microcolonies but produced very large flow-induced microcolonies, such that by only 8 h of growth Δ*mcm1*, Δ*tye7*, and Δ*rca1* cells formed microcolonies that were already the same size or larger than microcolonies formed by 18 h WT cells (Fig 5). The known functions of these *C*. *albicans* TRs are environmental sensing and metabolism [27,50]; however our results suggest they may have other roles in repression of filamentous growth induced by flow.

Core microcolony regulators Sfl1 and Sfl2 previously have been shown to bind and regulate numerous genes and TRs that play a role in biofilm formation and morphogenesis through ChIP-seq experiments [11,29]. Our work demonstrates the central involvement of Sfl1 and Sfl2 in the development of microcolonies, which is a novel function for this TR pair, as they were previously not identified as regulators of classical static biofilm formation [11,12]. This result combined with our gene expression data demonstrates that microcolonies are unique from a static biofilm model not only in their architecture, but also in their gene expression and regulation profiles.

Flow-induced microcolonies form as intertwining branching hyphal structures with extensive lateral budding yeast and pseudohyphae (Fig 1). These microcolony structures have several major differences compared with static *in vitro* biofilms that affect their inherent pathogenicity. First, microcolonies are substantially denser; and interestingly, we found that hyphae forming these microcolonies frequently grow in a sinusoidal growth pattern, an understudied pattern of growth that has been shown to be more resistant to antifungal treatment [51,52]. Secondly, hyphae in flow-induced microcolonies form buds that release from the mother cell to disperse from the biofilm throughout growth of the microcolony so that the process of dispersion is not a separate phase, but rather is concomitant with the growth of the microcolony (S1 Video). Thirdly, a large proportion of core microcolony genes expressed consist of fungal secreted proteins that have known roles in host damage including Ece1, Sap5, Hwp1 and Sod5. Thus flow has a major impact on not only the architecture of microcolonies, but also the genetic response of *C*. *albicans*. While further study of these structures is needed, our data indicate that flow-induced microcolonies are a specialized pathogenic form of biofilm that closely reflects biofilms formed on mucosal surfaces during oropharyngeal candidiasis.

## Materials and methods

### Strains and media

*Candida albicans* strains used in this study are shown in S3 Table. Cultures for all experiments were grown overnight in 1% (w/v) yeast extract, 2% (w/v) bacto peptone, and 2% (w/v) glucose (YPD; Difco, Detroit, MI). Complementation strains of *SFL1* (Δ*sfl1*/*SFL1*) and *ROB1* (Δ*rob1*/*ROB1*) genes were constructed by PCR and homologous recombination using vector pSN105 as described earlier [53].

### Flow system

The flow apparatus was configured as previously described [7]. Cell densities of overnight cultures were determined using a cytometer, and values were used to determine volumes of overnight culture to add to the attachment flask to reach 1 × 10^6^ cells/ml. After addition of culture, cells were allowed to acclimate for 15 min prior to initiation of flow. Flow experiments were split into two phases by using two separate media flasks. During the first phase (attachment phase), fresh YPD seeded with *C*. *albicans* cells (1 × 10^6^ cells/ml) was circulated through a μ-Slide I 0.8 Luer family ibiTreat flow chamber (ibidi, Martinsried, Germany) using a peristaltic pump. This phase proceeded for 2 hours, during which time cells were able to attach to the coverslip surface of the flow chamber. Afterwards, the source of media to the slide was switched to cell-free YPD for the remainder of the experiment (growth phase). The return flow during the growth phase was passed through four sequential cell filters, first two coarse filters (20 and 10 μm pore size; Analytical Scientific Instruments, Richmond, CA), then a 2 μm pore HPLC filter (Sigma Aldrich, St. Louis, MO) followed by a 0.22 μm PVDF filter (Sterivex; Millipore, Billerica, MA), before being recycled so as to prevent contamination of the stock medium. Thus, during the attachment phase, cells are allowed to re-circulate across the surface of the slide, but during the growth phase all cells are removed prior to re-circulation, and media to the slide remains cell free for the rest of the experiment.

In all experiments the flow was set to generate a shear force of 0.8 dynes/cm^2^ across the surface of the flow chamber. This value has been previously calculated as the approximate shear force that human saliva exerts on the tooth surface [54]. A hotplate stirrer with an external temperature probe was used to warm the media to 37°C. Additionally, the microscope, including the slide being imaged and several feet of preceding tubing, were warmed to 37°C, maintaining biofilm growth at this temperature.

Images were taken as previously described [7], using a Zeiss AxioScope A.1 transmitted light microscope with darkfield illumination. Images were acquired every two minutes during the attachment phase, and every 15 minutes during the growth phase.

### Static microcolony formation

Microcolony formation was examined as described previously [6]. For induction of microcolonies, overnight cultures of wild type and deletion strains were washed twice with 1 × phosphate buffered saline (PBS; Thermo Fisher Scientific, Waltham, MA), and diluted to 1 × 10^3^ cells/ml in PBS. A total of 100 μl of *C*. *albicans* cells was inoculated into 1ml RPMI medium (Thermo Fisher Scientific) per well of a 12-well cell culture plate (Corning Inc., Corning, NY) and incubated at 37°C for 24h in 5% CO_2_. Microcolonies were observed using brightfield microscopy with a Zeiss Axio Observer Z1 microscope (Zeiss, Göttingen, Germany) at 10 × magnification. The microcolony density was calculated using ImageJ software [55]. Briefly, a sliding paraboloid background subtraction was applied to image negatives with a radius of 225 microns. Then, automatic thresholding was applied using the Renyi Entropy method to outline the microcolonies [56]. A Gaussian blur with a sigma value of 10 was applied to correct for gaps that occurred following thresholding. The resulting contiguous regions of at least 1000 square microns were used to evaluate the integrated pixel value density in the corresponding microcolony of the unprocessed negative. This value was divided by the area of the microcolony to determine the density per μm^2^. Statistical significance was evaluated by one-way ANOVA with a post-hoc Tukey’s multiple comparison test (*p* < 0.05 significance level).

### RNA isolation

*Candida* cells were grown on an ibidi ibitreat flow chamber for 24 h at 23°C or 37°C under flow, or on an ibidi ibitreat 35mm μ-Dish at 37°C with gentle agitation (80 rpm on shaker). The bottom of the slide or dish was cut with a razor blade, removed, and placed immediately in 1 × PBS. The cells were removed by sonication from the slide (power cycle 4, 20% pulse rate for 1 m) and were collected by centrifugation at 2300 × g for 5 min. The cell pellet was re-suspended again in 1ml of Trizol and vortexed (4 cycle, 6m/s) with 0.45 μM glass beads using a FastPrep-24 5g Homogenizer (MP Biomedicals, Santa Ana, CA). After vortexing, 200μl of chloroform was added to the lysed cells, mixed vigorously for 15 sec and kept for 2 min at room temperature. Lysed cells were centrifuged at 21000 × g for 10 min at 4°C to separate the upper clear aqueous layer and mixed with 0.5 volume of 100% ethanol to precipitate total RNA from *Candida* cells. The total RNA was further purified using RNAeasy minikit from Qiagen (Hilden, Germany) according to manufacturer’s instructions. The RNA samples were stored at −80^o^ C.

### SYBR Green-based quantitative RT-PCR

Total RNA from *C*. *albicans* WT and TR mutants Δ*sfl1* (flow and static), Δ*sfl2* (flow and static) and Δrob1 (static) were isolated from microcolonies as described above to quantitate gene expression of selected core microcolony genes (*HWP1*, *HYR1*, *ECE1*, *PGA10*, *SAP5* and *HGC1*). Total cDNA was synthesized for each sample using an iScript cDNA synthesis kit (Bio-Rad) following the manufacturer’s instructions, with equal amounts of RNA (1 μg in a 20 μl reaction mixture). All samples were prepared with iQ SYBR Green Supermix (Bio-Rad), using 150 nM of both forward and reverse primers, and 1 μl of cDNA template in 20μl reactions. Primers used were *ACT1*qRTForward: ACTGCTTTGGCTCCATCTTCT; *ACT1*qRTReverse: TGGATGGACCAGATTCGTCG; *ECE1*qRTForward: TTGCTAATGCCGTCGTCAGA; *ECE1*qRTReverse: CCAGGACGCCATCAAAAACG; *HWP1*qRTForward: CTCCTGCCACTGAACCTTCC; *HWP1*qRTReverse: GAGCCAGCTGGAGCAGTTT; *HYR1*qRTForward: GCTGCTGCCCTTCCACAATA; *HYR1*qRTReverse: GGTGCAGATGGTCCATTGGT; *PGA10*qRTForward: GGTGTCGGGGAACCATACTG; PGA10qRTReverse: GGAGGTAGTGGCAAGCTCAG; *SAP5*qRTForward: ATTTCCCGTCCATGAGACTG; *SAP5*qRTReverse: ACCACGCCATTTTGGAATAC; *HGC1*qRTForward: CACCACCACAAATGCATTCTCA; *HGC1*qRTReverse: ATGAGGTGCAGGAAGCTGAC. After an initial denaturing step of 3 min at 95°C, the reactions were cycled 40 times under the following parameters: 95°C for 30 s, 55°C for 30 s, and 72°C for 30 s using CFX-96 Touch Real Time PCR system (Bio-Rad). Each reaction mixture contained negative RNA controls, no-template controls, and positive genomic DNA controls. Fluorescent data were collected and analyzed with iCycler iQ software. The standard curve method was employed for relative quantification using *ACT1* as control. Relative fold changes in gene expression of genes of interest (*HWP1*, *HYR1*, *ECE1*, *PGA10*, *SAP5* and *HGC1*) were calculated from the corresponding standard curves compared to *ACT1*. Data presented in the graph is the mean of three replicates taken from at least two independent experiments, with error bars representing standard differences between replicates.

### Library construction and RNA-Sequencing

Total RNA was quantified using Ribogreen Assay (Invitrogen, Carlsbad, CA) and the quality of RNA samples validated using Fragment Analyzer Standard Sensitivity Assay (Advanced Analytical, Ankeny, IA). Illumina TruSeq RNA sample preparation kit (Illumina, San Diego, CA) was used to prepare cDNA libraries from RNA samples as per manufacturer’s instructions. The cDNA libraries were quantified using Picogreen Assay (Invitrogen) and Library Quantification kit (Kappa Biosciences, Oslo, Norway). Fragment Analyzer High Sensitivity NGS kit (Advanced Analytical) was used to confirm the quality and size of the cDNA libraries. The cDNA libraries were then normalized, pooled and sequenced using the Illumina HiSeq 2500 at the UB Genomics and Bioinformatics Core Facility (Buffalo, NY). Sequencing reads were annotated and aligned to *C*. *albicans* reference genome (SC5314) using TopHat2 followed by Cufflinks to calculate the gene expressions for each gene in each sample and fold changes in gene expression among samples was determined using Cuffdiff [57]. A gene was considered differentially expressed if the false discovery rate for differential expression was P< 0.05. These data have been deposited in NCBI’s Gene Expression Omnibus [58] and are accessible through GEO Series accession number GSE117433 (https://www.ncbi.nlm.nih.gov/geo/query/acc.cgi?acc=GSE117433).

### Epithelial cells

TR146 buccal epithelial squamous cell carcinoma line was obtained from European Collection of Authenticated Cell Cultures (ECACC). TR146 cells were routinely cultured in 1:1 DMEM/F-12 medium supplemented with 10% FBS and maintained at 37°C in a 5% CO_2_ humidified incubator. For standard experiments, TR146 epithelial cells were seeded at 1×10^5^ cells/ml on acid washed 15 mm diameter glass coverslips previously placed in 12 well cell culture plates and cultured until the cells were confluent.

### *C*. *albicans* adhesion to epithelial cells

Adhesion assays were performed using TR146 monolayer epithelial cells as described previously [31]. Briefly, TR146 oral epithelial cells were grown to confluence on 15mm glass coverslips for 48 h in 12 well tissue culture plates, and were serum starved overnight prior to experiment. For adhesion, *Candida* cells (1 × 10^5^) were added into 1 ml serum-free DMEM/F-12 medium and incubated for 90 min that allows >90% of cell adhesion. After incubation, non-adherent *C*. *albicans* cells were removed and washed three times with 1 × PBS and fixed with 4% formaldehyde. After fixation, epithelial cells were permeabilized (0.1% Triton X-100 in PBS) for 15 min and adherent *Candida* cells were stained. For staining, *Candida* cells were incubated with rabbit anti-*Candida* antibody (1:1000 in 1 × PBS; OriGene, Rockville, MD) for 2 h and subsequently with a goat anti-rabbit-Alexa Fluor 488 antibody (1:2000 in 1 × PBS; Thermo Fisher Scientific) for 1h at 23°C and documented using Zeiss Axio Observer Z.1 microscope. The number of adherent cells was determined by counting 200 high-magnification fields of 150 μm × 150 μm size. The percentage of adherent cells in deletion strains were calculated compared to wild type adherent cells.

### *C*. *albicans* epithelial invasion assay

The number of *C*. *albicans* cells that invaded epithelial cells was determined as previously described with some modifications [31]. Monolayers of TR146 epithelial cells were grown on 15 mm diameter glass coverslips to confluence (2 days). The TR146 cells were infected with 1 × 10^5^ log phase *Candida* cells and placed in a humidified incubator for 4.5 h(sufficient for >80% invading hyphae). After 4.5 h incubation, the medium covering the cells was aspirated and monolayers were washed three times with 1 × PBS to remove non-adherent fungal cells. Next, epithelial cells were fixed with 4% formaldehyde for 10–15 min at 23°C. Non-invading adherent candida cells were stained using anti-candida antibody as described above. After staining, TR146 monolayer cells were permeabilized using 0.1% Triton X-100 for 20 min at 37°C in the dark. After permeabilization, total *C*. *albicans* cells were stained with 1mg/ml Calcofluor White (Sigma Aldrich) for 20 min at 20°C in the dark with gentle shaking. The coverslips were rinsed in water and mounted on slides using 1–2 drops of fluorescent mounting medium (Agilent, Santa Clara, CA) and allowed to air-dry for 1–2 h in the dark. Fluorescence imaging was performed using a Zeiss Axio Observer Z.1 microscope. The percentage of invading *C*. *albicans* cells was determined by dividing the number of invading cells by the total number of adherent cells. At least 200 *C*. *albicans* cells were counted to calculate percentage invasion.

### Epithelial cell damage assay

Epithelial cell damage induced by *C*. *albicans* microcolonies formed by WT and TR deletion strains was determined by release of lactate dehydrogenase (LDH) from epithelial cells following 24 h incubation using the Cytotoxicity Detection Kit (Cayman Chemical). For these assays, TR146 epithelial cells were seeded in 96 well tissue culture plates (Corning Inc, USA) and allowed to grow 48 h to 95% confluency. Confluent epithelial cells were washed twice with Dulbecco’s Phosphate-Buffered Saline (DPBS, Gibco) and serum free DMEM/F-12 medium was added prior to infection. Cultures of *C*. *albicans* WT and microcolony TR deletion strains grown overnight at 30°C in YPD (BD Biosciences) were washed in PBS, and approximately 1 × 10^3^ *C*. *albicans* cells were added to each well. *Candida* and epithelial cells were co-incubated for 24 h at 37°C in 5% CO_2_, then the plate was centrifuged at 500 x g for 5 min, and 100 μl of the culture supernatant from each well was collected. LDH released was measured at A_492_ using a Flexstation3 Multi-Mode Microplate Reader (Molecular Devices). Uninfected TR146 cells in DMEM/F-12 medium and C. *albicans* alone were used as controls. Uninfected TR146 cells in DMEM/F-12 medium supplemented with 1% Triton X-100 for 1 h were used as a positive control. Each experiment was performed in triplicate and at least twice, and differences between experimental groups were evaluated for significance using One-way ANOVA.

### Protein isolation and Western blotting

Total proteins from *Candida* infected epithelial cells were isolated as previously described with modifications [31]. TR146 epithelial cells were infected with each *Candida* strain at MOI (1:10). The *Candida* cells were allowed to infect epithelial cells for 3 h. After infection, the epithelial cells were lysed using 400μl RIPA buffer (50 mM Tris-HCl, pH 7.4, 150 mM NaCl, 1 mM EDTA, 1% Triton X-100, 1% sodium deoxycholate, 0.1% SDS) containing complete protease inhibitor cocktail (Roche, Basel, CH) and phosphatase inhibitors, left on ice for 30 min, then centrifuged for 10 min at 21000 × g at 4°C. Total protein concentration was estimated using bicinchoninic acid (BCA) assay (Thermo Fisher Scientific) and stored at −80°C. For immunoblotting, total protein (30μg) was separated by 12% SDS-PAGE and transferred to nitrocellulose membrane. Membranes were blocked in 5% milk or Bovine Serum albumin (BSA) in TBS containing 0.1% Tween-20 (TBST) at 23°C for 1 h. Blots were incubated in primary antibody (Phospo-MKP1 [S359] and c-Fos rabbit monoclonal antibodies (Cell Signaling Technology, Danvers, MA) for 16 h at 4°C. Membranes were washed twice with TBST and probed with a secondary antibody for 1 h at 23°C. Secondary antibodies were detected using SuperSignal West Pico detection kit (Thermo Fisher Scientific).

## Supporting information

S1 Fig Genes affecting microcolony formation. (A) Homozygous knockout mutants and wild-type CAI4 cells were grown under static microcolony inducing conditions (RPMI with 5% CO_2_) for 20 h, and imaged using brightfield microscopy. Images shown have significantly reduced microcolony density, except Δ*lip8*. Scale bar indicates 100 μm. (B) Microcolony density per square micron was evaluated using ImageJ. Data are means ± SD of n≥3 experiments, with * indicating significance by a post-hoc Tukey’s test at *p* < 0.05 as compared to WT.
(PDF)

S2 Fig Ten identified primary regulators of the core microcolony genes. The 20 core microcolony genes (left) were analyzed using the PathoYeastract database to predict transcriptional regulators (TR) that regulated at least half of the core microcolony genes (right). Arrows indicate regulatory associations between TR and their target genes. Color indicates either the number of TR a core microcolony gene is regulated by (red to yellow), or the number of microcolony genes that a TR regulates (yellow to green).
(PDF)

S3 Fig Transcription factors affecting microcolony formation. (A) Homozygous knockout mutants and wild-type CAI4 cells were grown under static microcolony inducing conditions (RPMI with 5% CO_2_) for 20 h, and imaged using brightfield microscopy. Images shown have significantly reduced microcolony density, except Δ*ofi1* and Δ*bcr1*. Scale bar indicates 100 μm. (B) Microcolony density per square micron was evaluated using ImageJ. Data are means ± SD of n≥3 experiments, with * indicating significance by a post-hoc Tukey’s test at *p* < 0.05 as compared to WT.
(PDF)

S4 Fig Several core microcolony genes are involved in microcolony adhesion or invasion. *Candida albicans* knockouts of eight transcriptional regulators were quantitated for adhesion (90 min incubation) and invasion (4.5 h) on TR146 epithelial monolayers and compared to wild-type CAI4 cells. For adhesion and invasion, non-adherent *C*. *albicans* cells were removed by washing, and adherent cells fixed with 4% formaldehyde. For invasion, epithelial cells were also permeabilized and adherent *Candida* cells were stained with anti-*Candida* antibody and Alexa Fluor 488. Asterisks indicate statistically significant differences compared to WT cells, * p<0.05, ** p<0.01, *** p<0.001. ND: No data.
(PDF)

S1 Table (A) RNA-seq transcriptomic data of C. albicans microcolonies grown at 37°C under flow as compared to cells grown at 37°C statically (B) RNA-seq transcriptomic data of *C*. *albicans* microcolonies grown at 37°C under flow as compared to cells grown at 23°C under flow
(XLSX)

S2 Table Pathoyeastract predicted transcriptional factor (TF) dataset. Core microcolony genes were used to predict potential transcriptional factors. Analysis performed on July 13th, 2017.
(XLSX)

S3 Table Strains used in the study. All deletion strains used were homozygous knockouts.
(DOCX)

S1 Video Microcolony formation of *C*. *albicans* WT cells under flow at 37°C. This time-lapse darkfield microscopy video shows the attachment of WT cells to the substrate during the attachment phase (time indicated in the upper left hand corner; images acquired every 2 min), followed by the subsequent growth and development of the biofilm during the growth phase (starts at 2 h; images acquired every 15 min). Cell-seeded media (1×10^6^) was used during the attachment phase, while cell-free media was used during the growth phase. Flow is from the right to left. Scale bar indicates 100 μm.
(WMV)

S2 Video *C*. *albicans* Δ*efg1* cells do not form biofilm under flow. This time-lapse darkfield microscopy video shows the attachment of Δ*efg1* cells to the substrate during the attachment phase (time indicated in the upper left hand corner; images acquired every 2 min), followed by the growth phase (starts at 2 h; images acquired every 15 min), where the cells failed to remain adhered over time. Cell-seeded media (1×10^6^) was used during the attachment phase, while cell-free media was used during the growth phase. Flow is from the right to left. Scale bar indicates 100 μm.
(WMV)

S3 Video *C*. *albicans* Δ*rob1* cells form small microcolonies under flow. This time-lapse darkfield microscopy video shows the attachment of Δ*rob1* cells to the substrate during the attachment phase (time indicated in the upper left hand corner; images acquired every 2 min), followed by the subsequent growth and development of the biofilm during the growth phase (starts at 2 h; images acquired every 15 min). Cell-seeded media (1×10^6^) was used during the attachment phase, while cell-free media was used during the growth phase. Flow is from the right to left. Scale bar indicates 100 μm.
(WMV)

S4 Video *C*. *albicans* Δ*ndt80* cells do not form biofilm under flow. This time-lapse darkfield microscopy video shows the attachment of Δ*ndt80* cells to the substrate during the attachment phase (time indicated in the upper left hand corner; images acquired every 2 min), followed by the growth phase (starts at 2 h; images acquired every 15 min), where the cells failed to remain adhered over time. Cell-seeded media (1×10^6^) was used during the attachment phase, while cell-free media was used during the growth phase. Flow is from the right to left. Scale bar indicates 100 μm.
(WMV)

S5 Video *C*. *albicans* Δ*sfl1* cells form slightly larger microcolonies under flow. This time-lapse darkfield microscopy video shows the attachment of Δ*sfl1* cells to the substrate during the attachment phase (time indicated in the upper left hand corner; images acquired every 2 min), followed by the subsequent growth and development of the biofilm during the growth phase (starts at 2h; images acquired every 15 min). Cell-seeded media (1×10^6^) was used during the attachment phase, while cell-free media was used during the growth phase. Flow is from the right to left. Scale bar indicates 100 μm.
(WMV)

S6 Video *C*. *albicans* Δ*sfl2* cells form microcolonies under flow, but are substantially delayed compared to WT. This time-lapse darkfield microscopy video shows the attachment of Δ*sfl2* cells to the substrate during the attachment phase (time indicated in the upper left hand corner; images acquired every 2 min), followed by the subsequent growth and development of the biofilm during the growth phase (starts at 2 h; images acquired every 15 min). Cell-seeded media (1×10^6^) was used during the attachment phase, while cell-free media was used during the growth phase. Flow is from the right to left. Scale bar indicates 100 μm.
(WMV)

S7 Video *C*. *albicans* Δ*mcm1* cells form more dense microcolonies under flow. This time-lapse darkfield microscopy video shows the attachment of Δ*mcm1* cells to the substrate during the attachment phase (time indicated in the upper left hand corner; images acquired every 2 min), followed by the subsequent growth and development of the biofilm during the growth phase (starts at 2 h; images acquired every 15 min). Cell-seeded media (1×10^6^) was used during the attachment phase, while cell-free media was used during the growth phase. Flow is from the right to left. Scale bar indicates 100 μm.
(WMV)

S8 Video *C*. *albicans* Δ*tye7* cells form more dense microcolonies under flow. This time-lapse darkfield microscopy video shows the attachment of Δ*tye7* cells to the substrate during the attachment phase (time indicated in the upper left hand corner; images acquired every 2 min), followed by the subsequent growth and development of the biofilm during the growth phase (starts at 2 h; images acquired every 15 min). Cell-seeded media (1×10^6^) was used during the attachment phase, while cell-free media was used during the growth phase. Flow is from the right to left. Scale bar indicates 100 μm.
(WMV)

S9 Video *C*. *albicans* Δ*rca1* cells form more dense microcolonies under flow. This time-lapse darkfield microscopy video shows the attachment of Δ*rca1* cells to the substrate during the attachment phase (time indicated in the upper left hand corner; images acquired every 2 min), followed by the subsequent growth and development of the biofilm during the growth phase (starts at 2 h; images acquired every 15 min). Cell-seeded media (1×10^6^) was used during the attachment phase, while cell-free media was used during the growth phase. Flow is from the right to left. Scale bar indicates 100 μm.
(WMV)
