# Supplementary material for: Candida albicans Sfl1/Sfl2 regulatory network drives the formation of pathogenic microcolonies
Source: PLoS Pathog. 2018 Sep 25;14(9):e1007316. doi: 10.1371/journal.ppat.1007316 (PMC6173444; doi:10.1371/journal.ppat.1007316)
Supplement: S1 Fig — (A) Homozygous knockout mutants and wild-type CAI4 cells were grown under static microcolony inducing conditions (RPMI with 5% CO2) for 20 h, and imaged using brightfield microscopy. Images shown have significantly reduced microcolony density, except Δlip8. Scale bar indicates 100 μm. (B) Microcolony density per square micron was evaluated using ImageJ. Data are means ± SD of n≥3 experiments, with * indicating significance by a post-hoc Tukey’s test at p < 0.05 as compared to WT. (PDF) [file ppat.1007316.s001.pdf]

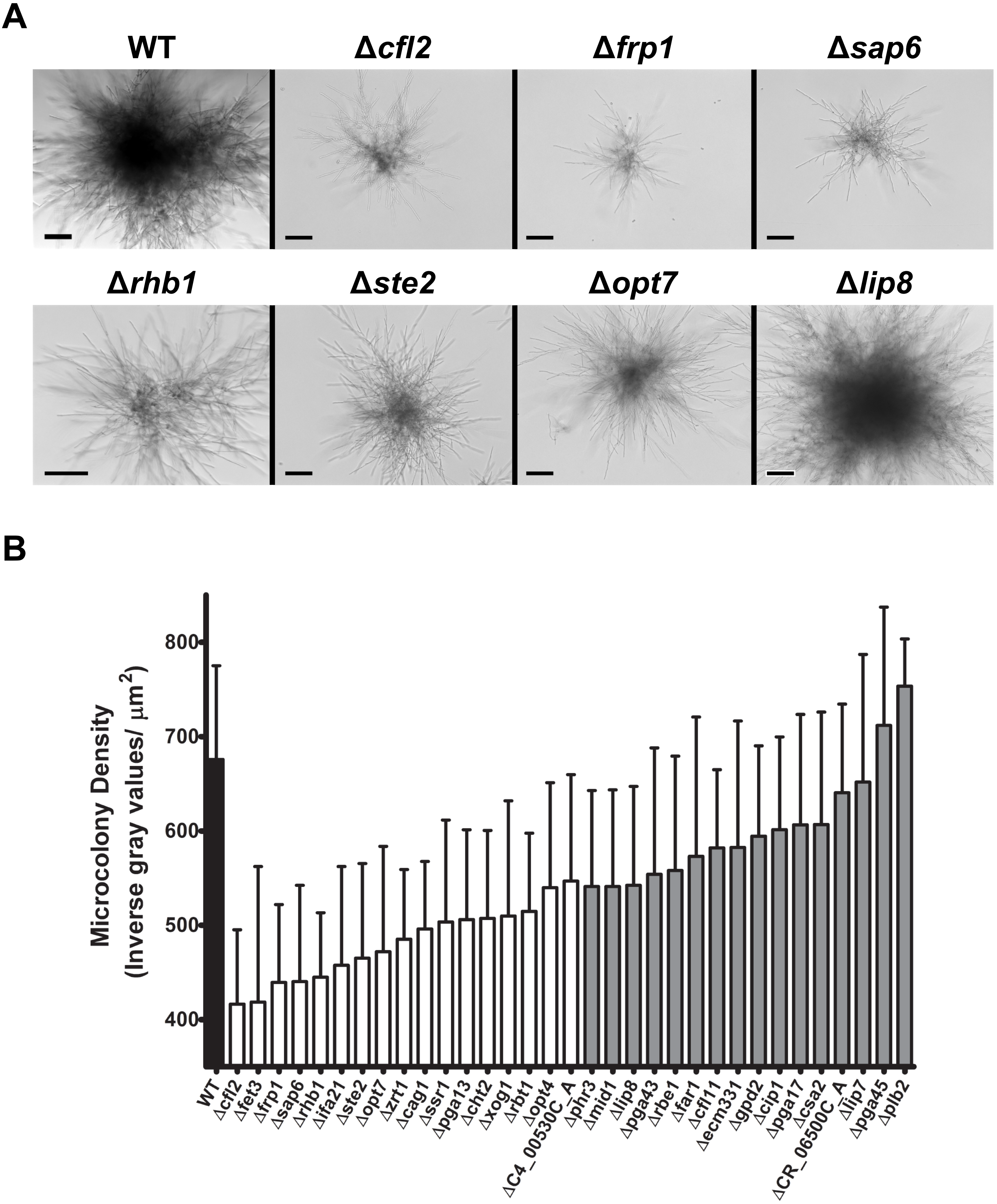

S1 Fig. Genes affecting microcolony formation.

(A) Homozygous knockout mutants and wild-type CAI4 cells were grown under static microcolony inducing conditions (RPMI with 5% CO_2) for 20 h, and imaged using brightfield microscopy. Images shown have significantly reduced microcolony density, except Δ*lip8*. Scale bar indicates 100 μm. (B) Microcolony density per square micron was evaluated using ImageJ. Data are means ± SD of n≥3 experiments, with white bars indicating significance by a post-hoc Tukey's test at $p < 0.05$ as compared to WT.
