# Supplementary material for: Candida albicans Sfl1/Sfl2 regulatory network drives the formation of pathogenic microcolonies
Source: PLoS Pathog. 2018 Sep 25;14(9):e1007316. doi: 10.1371/journal.ppat.1007316 (PMC6173444; doi:10.1371/journal.ppat.1007316)
Supplement: S2 Fig — The 20 core microcolony genes (left) were analyzed using the PathoYeastract database to predict transcriptional regulators (TR) that regulated at least half of the core microcolony genes (right). Arrows indicate regulatory associations between TR and their target genes. Color indicates either the number of TR a core microcolony gene is regulated by (red to yellow), or the number of microcolony genes that a TR regulates (yellow to green). (PDF) [file ppat.1007316.s002.pdf]

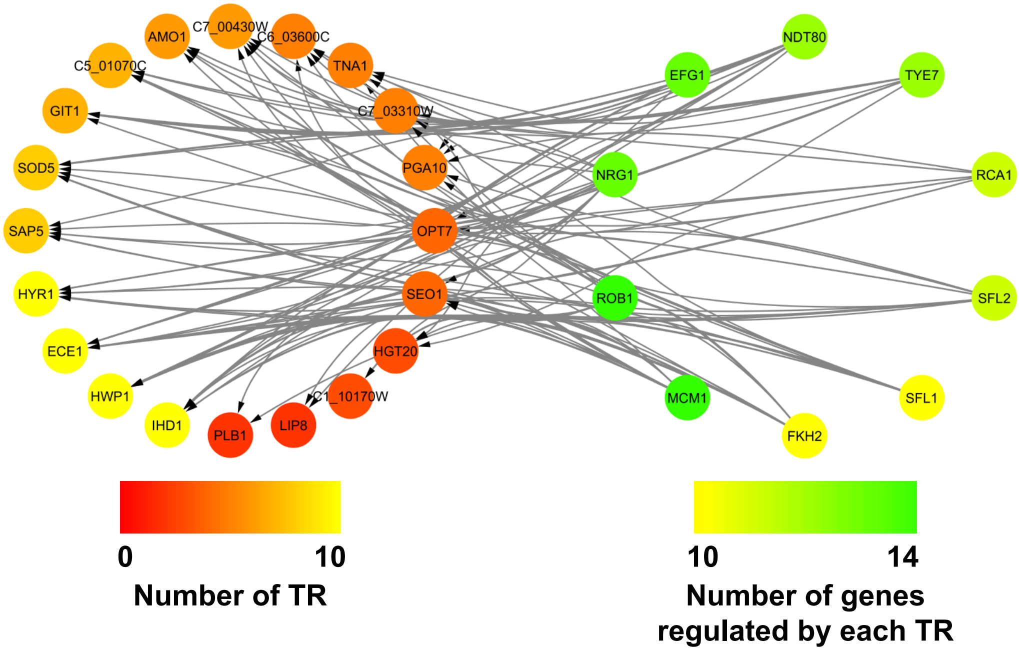

S2 Fig. Ten identified primary regulators of the core microcolony genes. The 20 core microcolony genes (left) were analyzed using the Pathoyeastract database to predict transcriptional regulators (TR) that regulated at least half of the core microcolony genes (right). Arrows indicate regulatory associations between TR and their target genes. Color indicates either the number of TR a core microcolony gene is regulated by (red to yellow), or the number of microcolony genes that a TR regulates (yellow to green).
