# Supplementary material for: Candida albicans Sfl1/Sfl2 regulatory network drives the formation of pathogenic microcolonies
Source: PLoS Pathog. 2018 Sep 25;14(9):e1007316. doi: 10.1371/journal.ppat.1007316 (PMC6173444; doi:10.1371/journal.ppat.1007316)
Supplement: S4 Fig — Candida albicans knockouts of eight transcriptional regulators were quantitated for adhesion (90 min incubation) and invasion (4.5 h) on TR146 epithelial monolayers and compared to wild-type CAI4 cells. For adhesion and invasion, non-adherent C. albicans cells were removed by washing, and adherent cells fixed with 4% formaldehyde. For invasion, epithelial cells were also permeabilized and adherent Candida cells were stained with anti-Candida antibody and Alexa Fluor 488. Asterisks indicate statistically significant differences compared to WT cells, * p<0.05, ** p<0.01, *** p<0.001. ND: No data. (PDF) [file ppat.1007316.s004.pdf]

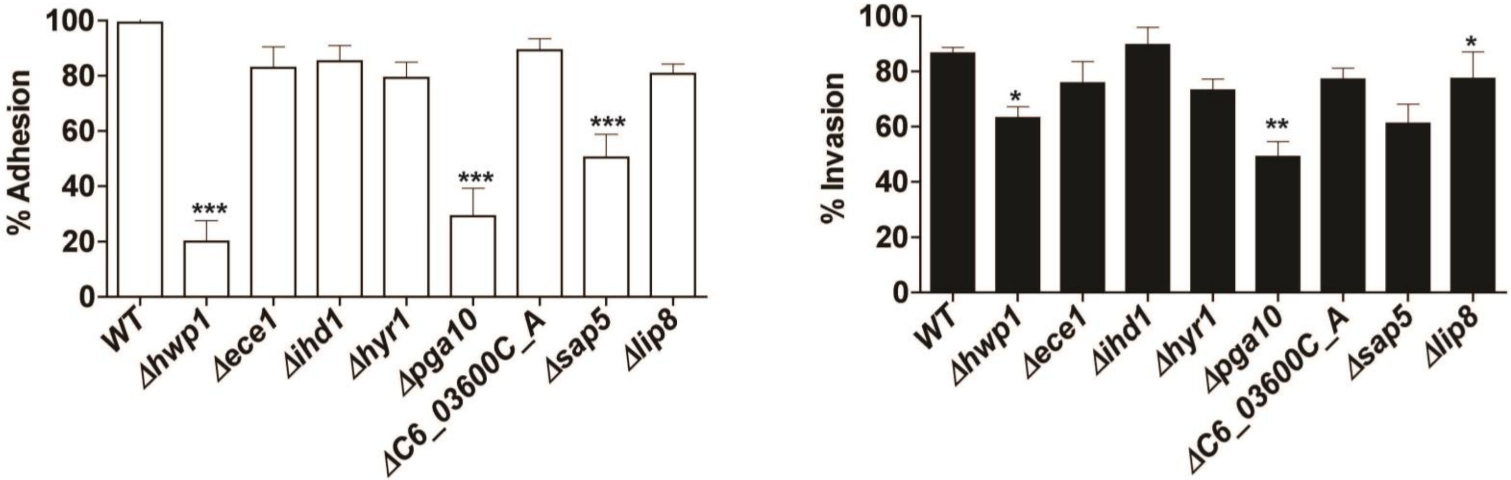


**S4 Fig. Several core microcolony genes are involved in microcolony adhesion or invasion.** *Candida albicans* knockouts of eight transcriptional regulators were quantitated for adhesion (90 min incubation) and invasion (4.5 h) on TR146 epithelial monolayers and compared to wild-type CAI4 cells. For adhesion and invasion, non-adherent *C. albicans* cells were removed by washing, and adherent cells fixed with 4% formaldehyde. For invasion, epithelial cells were also permeabilized and adherent *Candida* cells were stained with anti-*Candida* antibody and Alexa Fluor 488. Asterisks indicate statistically significant differences compared to WT cells, * $p<0.05$, ** $p<0.01$, *** $p<0.001$. ND: No data.
