# Supplementary material for: Candida albicans Sfl1/Sfl2 regulatory network drives the formation of pathogenic microcolonies
Source: PLoS Pathog. 2018 Sep 25;14(9):e1007316. doi: 10.1371/journal.ppat.1007316 (PMC6173444; doi:10.1371/journal.ppat.1007316)
Supplement: S3 Table — All deletion strains used were homozygous knockouts. (DOCX) [file ppat.1007316.s007.docx]

| **Name** | **Strain/Genotype** | **Reference** |
| --- | --- | --- |
| WT | CAI4 | [1] |
| Isogenic WT | SN250 | [2] |
| Δ*bcr1* | SN275 | [2] |
| ΔC4_00530C_A | SN86 | [2] |
| ΔC6_03600C_A | SN127 | [2] |
| ΔCR_06500C_A | SN11 | [2] |
| Δ*cag1* | SN684 | [2] |
| Δ*cfl2* | SN340 | [2] |
| Δ*cfl11* | SN274 | [2] |
| Δ*cht2* | SN670 | [2] |
| Δ*cip1* | SN1 | [2] |
| Δ*csa2* | SN68 | [2] |
| Δ*cup9* | SN991 | [2] |
| Δ*crz1* | SN1092 | [2] |
| Δ*ece1* | SN72 | [2] |
| Δ*ecm331* | C180-5 | [3] |
| Δ*efg1* | TF156-X | [4] |
| Δ*far1* | SN1058 | [2] |
| Δ*fet3* | SN712 | [2] |
| Δ*fkh2* | YJB6292 | [5] |
| Δ*frp1* | SN897 | [2] |
| Δ*gdp2* | SN270 | [2] |
| Δ*hwp1* | FJS24 | [6] |
| Δ*hyr1* | SN823 | [2] |
| Δ*ifa21* | SN837 | [2] |
| Δ*ihd1* | SN917 | [2] |
| Δ*lip7* | SN1084 | [2] |
| Δ*lip8* | SN27 | [2] |
| Δ*mcm1* | CJN1007 | [3] |
| Δ*mid1* | SN585 | [2] |
| Δ*ndt80* | TF095-X | [4] |
| Δ*nrg1* | SN1063 | [2] |
| Δ*ofi1* | SN822 | [2] |
| Δ*opt4* | SN208 | [2] |
| Δ*opt7* | SN905 | [2] |
| Δ*pga10* | SN126 | [2] |
| Δ*pga13* | SN982 | [2] |
| Δ*pga17* | E592-1 | [7] |
| Δ*pga43* | SF050 | [8] |
| Δ*pga45* | SN499 | [2] |
| Δ*phr1* | SN660 | [2] |
| Δ*phr3* | FJS48 | [6] |
| Δ*plb2* | JJH13 | [8] |
| Δ*rbe1* | SN1070 | [2] |
| Δ*rbt1* | EA41-1 | [3] |
| Δ*rca1* | TF058-X | [4] |
| Δ*rhb1* | SN942 | [2] |
| Δ*rob1* | TF110-X | [4] |
| Δ*sap5* | SN893 | [2] |
| Δ*sap6* | SN886 | [2] |
| Δ*sfl1* | TF001-X | [4] |
| Δ*sfl2* | TF020-X | [4] |
| Δ*sfu1* | SN811 | [2] |
| Δ*ste2* | SN272 | [2] |
| Δ*ssr1* | FJS35 | [6] |
| Δ*tye7* | SN819 | [2] |
| Δ*wor2* | SN941 | [2] |
| Δ*wsc1* | D120-1 | [3] |
| Δ*wsc4* | BC40-3 | [3] |
| Δ*xog1* | SN1134 | [2] |
| Δ*zcf1* | SN222 | [2] |
| Δ*zrt1* | SN570 | [2] |
| Δ*rob1*/*ROB1* | *ura3*∆::*λimm434*::*URA3*-*IRO1* *arg4*::*hisG* *his1*::*hisG* *leu2*::*hisG*::*ROB1*-Cd*ARG4* *robΔ*::Cm*LEU2*  *ura3*∆::*λimm434* *arg4*::*hisG* *his1*::*hisG* *leu2*::*hisG* *rob1*Δ::Cd*HIS1* | This Study |
| Δ*sfl1*/*SFL1* | *ura3*∆::*λimm434*::*URA3*-*IRO1* *arg4*::*hisG* *his1*::*hisG* *leu2*::*hisG*::*SFL1*-Cd*ARG4* *sfl1Δ*::Cm*LEU2*  *ura3*∆::*λimm434* *arg4*::*hisG* *his1*::*hisG* *leu2*::*hisG* *sfl1Δ*::Cd*HIS1* | This study |
| Δ*sfl2*/*SFL2* | CaMS58 | [9] |

**S3 Table. Strains used in the study.** All deletion strains used were homozygous knockouts.

**References**

1. Fonzi WA, Irwin MY. Isogenic strain construction and gene mapping in *Candida albicans*. Genetics. 1993;134: 717–28.

2. Noble SM, French S, Kohn LA, Chen V, Johnson AD. Systematic screens of a *Candida albicans* homozygous deletion library decouple morphogenetic switching and pathogenicity. Nat Genet. 2010;42: 590–8. doi:10.1038/ng.605

3. Nobile CJ, Mitchell AP. Regulation of cell-surface genes and biofilm formation by the *C. albicans* transcription factor Bcr1p. Curr Biol. 2005;15: 1150–5.

4. Homann OR, Dea J, Noble SM, Johnson AD. A phenotypic profile of the *Candida albicans* regulatory network. PLoS Genet. 2009;5: e1000783. doi:10.1371/journal.pgen.1000783

5. Bensen ES, Filler SG, Berman J. A forkhead transcription factor is important for true hyphal as well as yeast morphogenesis in *Candida albicans*. Eukaryotic Cell. 2002;1: 787–98. doi:10.1128/EC.1.5.787-798.2002

6. Nobile CJ, Nett JE, Andes DR, Mitchell AP. Function of *Candida albicans* adhesin Hwp1 in biofilm formation. Eukaryotic Cell. 2006;5: 1604–10.

7. Norice CT, Smith FJ, Solis N, Filler SG, Mitchell AP. Requirement for *Candida albicans* Sun41 in biofilm formation and virulence. Eukaryotic Cell. 2007;6: 2046–55.

8. Konstantinidou N, Morrissey JP. Co-occurence of filamentation defects and impaired biofilms in *Candida albicans* protein kinase mutants. FEMS Yeast Res. 2015;15. doi:10.1093/femsyr/fov092

9. Spiering MJ, Moran GP, Chauvel M, Maccallum DM, Higgins J, Hokamp K, et al. Comparative transcript profiling of *Candida albicans* and *Candida dubliniensis* identifies SFL2, a *C. albicans* gene required for virulence in a reconstituted epithelial infection model. Eukaryotic Cell. 2010;9: 251–65. doi:10.1128/EC.00291-09
